# MRC2-mediated collagen internalization is reduced in fibrotic lung fibroblasts and increased upon phenotypic dedifferentiation

**DOI:** 10.1172/jci.insight.201712

**Published:** 2026-05-19

**Authors:** Natalie M. Walker, Sean M. Fortier, Jennifer Speth, Steven K. Huang, Sergey Gutor, Timothy S. Blackwell, Marc Peters-Golden

**Affiliations:** Division of Pulmonary and Critical Care Medicine, University of Michigan Medical School, Ann Arbor, Michigan, USA.

**Keywords:** Cell biology, Pulmonology, Collagens, Extracellular matrix, Lysosomes

## Abstract

Idiopathic pulmonary fibrosis (IPF) is characterized by parenchymal scarring reflecting an imbalance between collagen deposition by myofibroblasts (MFs) and its turnover. Although collagen clearance is essential for fibrosis resolution, this process and its potential for therapeutic modulation in IPF are poorly understood. Here we evaluated internalization of degraded collagen and the role of its requisite endocytic receptor mannose receptor C-type 2 (MRC2), in lung tissue and MFs from patients with IPF and bleomycin-injured mice. Fibrotic human and murine lung tissue exhibited an accumulation of degraded collagen, highlighting a failure of its clearance. MFs from fibrotic lung demonstrated a reduced capacity to internalize extracellular degraded collagen, with a concomitant reduction in MRC2 expression and endolysosomal activity. Both diminished collagen uptake and MRC2 expression recovered to baseline levels during spontaneous resolution of bleomycin fibrosis. In vitro treatment of IPF or TGF-β–elicited MFs with a variety of mechanistically distinct agents known to effect phenotypic dedifferentiation restored defective collagen internalization. Although enhanced uptake was MRC2 dependent, it involved increased endolysosomal activity rather than increased MRC2 expression. These results implicate defective MRC2-dependent collagen internalization and endolysosomal function in MFs as important factors contributing to fibrosis that may be therapeutically targeted to promote resolution.

## Abstract 2

We identify collagen clearance by lung myofibroblasts as a dynamic and tunable process that is impaired in fibrosis but may be leveraged to promote resolution.

## Graphical abstract

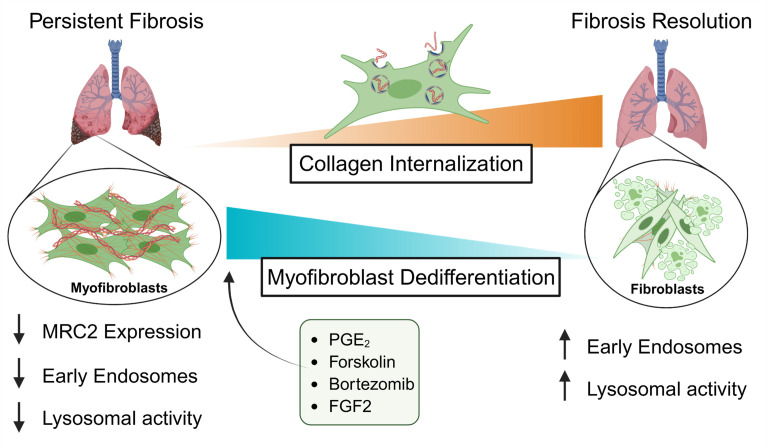

## Introduction

Idiopathic pulmonary fibrosis (IPF) is an intractable and progressive fibrotic lung disease of uncertain etiology (1, 2). A central feature is distorted alveolar architecture and eventual organ failure due to the accumulation of excessive collagen-rich, extracellular matrix (ECM) elaborated primarily by pathologic, apoptosis-resistant mesenchymal cells commonly termed myofibroblasts (MFs). Although collagen accumulation in IPF has been hypothesized to reflect inadequate degradation relative to its production, this concept is challenged by reports demonstrating increases in serum levels of collagen degradation products and in tissue or serum levels of various ECM-degrading matrix metalloproteinases (MMPs) in such patients (3–8). Additionally, studies in knockout mice have largely demonstrated that MMP loss confers protection against bleomycin-induced lung fibrosis (6, 7), further arguing against impaired degradation as the predominant determinant of a lack of collagen clearance in lung fibrosis.

Internalization and intracellular degradation of collagen fragments to clear ECM in the injured lung has largely been attributed to phagocytosis by macrophages (9–11). However, recent reports highlight the capability of fibroblasts to also engage in cellular internalization of degraded collagen, with a loss of this capacity in aging as a possible contributor to progression of pulmonary fibrosis (12). Despite its importance, the capacity of MFs from fibrotic lung tissue to internalize degraded collagen is largely unexplored.

While MFs were long considered to be terminally differentiated (13, 14), it is now apparent that they exhibit substantial plasticity (15). Indeed, in response to a number of mediators and pharmacologic agents (15–25), established MFs have been shown to globally downregulate their expression of contractile and fibrotic signature genes and proteins (16) — a phenomenon termed “dedifferentiation.” One critical accompanying feature of dedifferentiation is that MFs regain their sensitivity to apoptosis (16, 26). As MF apoptosis is required for the spontaneous resolution observed in young mice with bleomycin-induced fibrosis (26) and as in vitro MF dedifferentiation has been shown to distinguish between such resolution and the fibrosis persistence observed in aged mice (27), it is plausible that MF dedifferentiation serves as an in vitro correlate to fibrosis resolution in vivo. The fact that currently available drugs pirfenidone and nintedanib are incapable of dedifferentiating MFs (18) further supports this premise. Importantly, ECM clearance has received little attention in the context of either fibrosis resolution or MF dedifferentiation.

Here we report that fibrotic lung tissue from patients with IPF and bleomycin-treated mice demonstrated accumulation of degraded collagen species, and that MFs from fibrotic lung exhibited a diminished capacity for collagen fragment internalization as compared with that of normal lung fibroblasts. This defect was associated with diminished expression of the collagen endocytic receptor mannose receptor C-type 2 (MRC2), along with decreased endosomal vesicle formation and reduced lysosomal activity. Importantly, we found that resolution of fibrosis in vivo and in vitro dedifferentiation elicited by a variety of pharmacologically distinct agents promoted MF internalization of collagen fragments in a rapid and robust manner via mobilization of the endolysosomal machinery. This impairment in collagen clearance by fibrotic MFs suggests its possible contribution to persistent fibrosis, while its reversibility highlights it as a potential target for promoting fibrosis resolution.

A portion of this work was previously presented in abstract form (28).

## Results

### Fibrotic lung tissue exhibits accumulation of degraded collagen.

**Figure 1:**
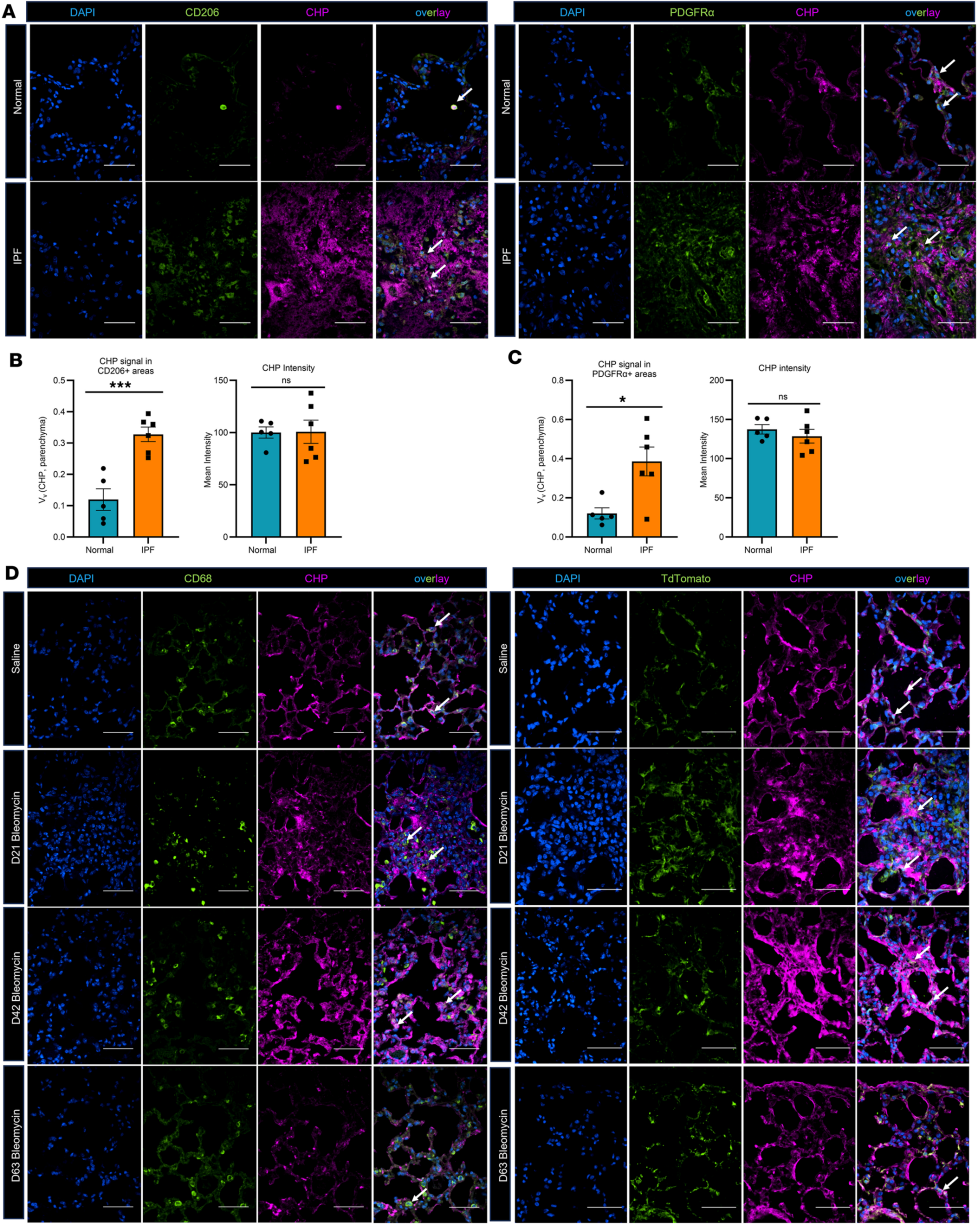
Degraded collagen is increased in IPF lung tissue and bleomycin-injured murine lungs. (**A**) Representative images of CHP fluorescent colocalization with CD206 (left panels) to label macrophages or PDGFR-α (right panels) to label fibroblasts in normal (top) and IPF (bottom) human lungs. In all figures, arrows denote colocalized collagen fragments and cell-specific markers. Quantified volumetric ratio of CHP to total parenchyma and mean intensity of CHP^+^ areas where (**B**) macrophages and (**C**) fibroblasts are present. (**D**) CHP fluorescent colocalization with CD68 (left panels) to label macrophages or tdTomato (right panels) to label fibroblasts in saline, day 21, day 42, and day 63 bleomycin-injured lungs. Images are representative of an *n* of 4 mice for each treatment group. Scale bars: 50 μm (**A** and **D**). Histomorphometric data points represent values from tissues of independent patients and significance in **B** and **C** was determined by 2-tailed *t* test. *P < 0.05, ***P < 0.001.

Numerous reports demonstrate elevated collagen degradation products in serum of IPF patients (3–5, 29, 30), and one report demonstrates elevated levels of degraded collagen in IPF tissues in situ (8). To determine the cellular localization of degraded collagen in lung tissues, we stained sections of normal and IPF lung with macrophage- and fibroblast-specific markers and fluorescently labeled collagen hybridizing peptide (CHP). CHP is a synthetic peptide that specifically binds to unwound, non–triple helical collagens. This specificity allows it to serve as a readout of the presence of a variety of forms of structurally denatured or degraded collagens, ranging in size from full-length monomers to large gelatin fragments to smaller degradation byproducts thereof termed collagen fragments (31, 32). It should be noted, however, that CHP staining does not distinguish among this panoply of degradation products. We performed CHP staining on formalin-fixed, paraffin-embedded lung sections in the absence of antigen retrieval, thus allowing for the capture of native, degraded collagen species in the tissue as previously described (8, 31, 33–35). Normal lung demonstrated minimal CHP staining, which was localized predominantly to alveolar and interstitial macrophages marked by CD206^+^ staining, with occasional staining localized to PDGFRα^+^ fibroblasts (Figure 1A, top panels). In contrast, IPF lung tissues demonstrated overall elevated CHP staining, with degraded collagen surrounding both CD206^+^ macrophages and PDGFRα^+^ fibroblasts (Figure 1A, bottom panels and Supplemental Figure 1, A and B; supplemental material available online with this article; https://doi.org/10.1172/jci.insight.201712DS1). Histologic density quantitation demonstrated a significant increase in the volumetric ratio of CHP staining to total parenchyma in IPF tissue areas containing CD206^+^ macrophage (Figure 1B) and PDGFRα^+^ fibroblast markers (Figure 1C), with no significant difference in the mean intensity of such staining noted. Peak fibrosis at 21 days after bleomycin injury was also characterized by elevated CHP staining in comparison with saline-treated lungs. CHP staining in fibrotic murine lungs was colocalized with and adjacent to CD68^+^ macrophages (Figure 1D). Col1a2Cre^+^tdTomato^+^ transgenic mice were used to label fibroblasts of the lung followed by CHP staining. tdTomato^+^ fibroblasts were found to be surrounded by CHP staining in many but not all areas (Figure 1D), demonstrating a heterogeneous contribution by fibroblasts to collagen clearance in situ. Our previous study utilizing the same mouse strain and bleomycin dosing strategy identified days 42 and 63 as representing half-maximal (~50%) and near-complete (~95%) fibrosis resolution, respectively (18). This time course of resolution was confirmed in a new experiment, representative trichrome images from which are shown in Supplemental Figure 2A. Bleomycin-injured lungs at day 42 after injury demonstrated fewer areas of CHP positivity, still surrounding macrophage- and fibroblast-specific markers, while CHP signal had returned back to baseline control levels at day 63 (Figure 1D). These data suggest that collagen degradation product accumulation increases with fibrosis and declines during the resolution process. Taken together, these data demonstrate an accumulation of degraded collagen products in tissues from IPF lungs and at peak fibrosis after bleomycin, with localization patterns suggesting contributions from both macrophage and fibroblast populations.

### Fibrotic MFs have decreased capacity to internalize gelatin, which is enhanced upon dedifferentiation and during fibrosis resolution.

**Figure 2:**
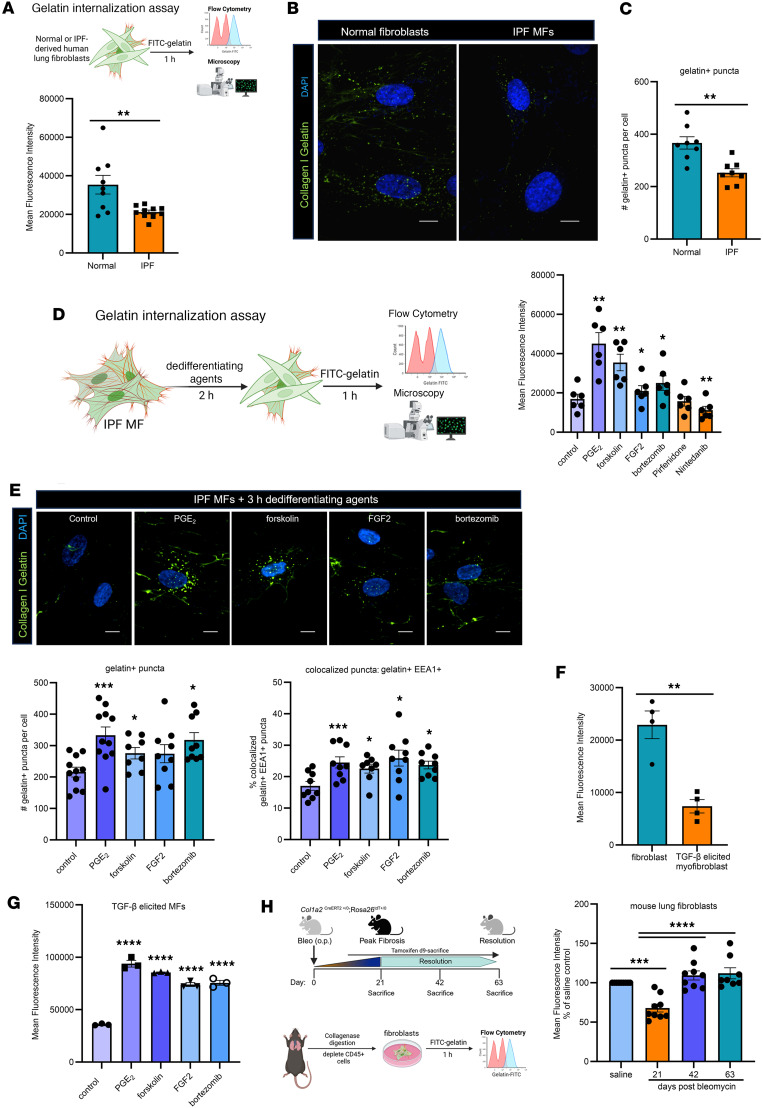
Fibrotic MFs demonstrate reduced gelatin internalization capacity that is enhanced upon treatment with dedifferentiation agents and upon fibrosis resolution. (**A**) Schematic demonstrating experimental protocol utilized for assessing gelatin internalization. Normal fibroblasts and IPF MFs were treated with Oregon Green 488–conjugated gelatin for 1 hour (5 μg/mL) followed by flow cytometric analysis. (**B**) Confocal images (×100, oil) demonstrating gelatin internalization in normal fibroblasts and IPF MFs, with gelatin^+^ puncta quantitated using CellProfiler (**C**). (**D**) Schematic demonstrating experimental protocol employed for assessing gelatin internalization after dedifferentiating agent treatment. IPF MFs were treated with dedifferentiating agents for 2 hours followed by addition of conjugated gelatin 1 hour prior to flow analysis for internalization. (**E**) Confocal imaging (×100) and gelatin puncta quantification along with EEA1^+^ early endosomal colocalization in IPF MFs after treatment with dedifferentiating agents and conjugated gelatin. Scale bars: 10 μm (**B** and **E**). Normal fibroblasts treated with TGF-β for 48 hours to establish MFs followed by flow cytometric analysis of gelatin internalization capacity at baseline (**F**) and (**G**) after treatment with dedifferentiating agents. (**H**) Schemata demonstrating bleomycin injury model timeline and experimental protocol used to assess fibroblasts’ gelatin internalization over time. Mouse fibroblasts isolated and grown in vitro from murine lungs of saline or bleomycin-injured mice at time points indicated were passaged 3 times, then treated with conjugated gelatin (5 μg/mL) for 1 hour and internalization capacity determined by flow cytometry. Each individual data point in **A** and **C**–**E** is derived from independent patient cell lines. Significance for **A**, **C**, and **F** was determined by 2-tailed *t* test, while a 1-way ANOVA was used in **D**, **E**, **G**, and **H** followed by Dunnett’s multiple comparisons test (**D**, **E**, and **G**) and Šidák’s multiple comparisons test (**H**). *P < 0.05; **P < 0.01; ***P < 0.001; ****P < 0.0001.

The accumulation of degraded collagen species surrounding fibroblasts in fibrotic tissues (Figure 1, A, C, and D) led us to consider whether impaired cellular internalization of collagen fragments by MFs might contribute to ECM accumulation in IPF. To determine the capacity of IPF and normal fibroblasts to internalize degraded collagen fragments, flow cytometry and confocal microscopy–based methods were employed to assess cellular internalization of exogenous Oregon Green 488–conjugated gelatin. Flow cytometry revealed an approximately 40% decrease in the ability of IPF MFs to internalize gelatin over a 1-hour interval as compared with normal fibroblasts (Figure 2A), and this was substantiated by a comparably reduced number of gelatin^+^ puncta per cell (Figure 2B) quantitated (Figure 2C) via confocal microscopy imaging and CellProfiler pipeline analysis, as previously described (36, 37). This defect in IPF MFs was sustained over a more protracted time course, with 3-, 6-, and 24-hour time points demonstrating an approximately 50% reduction in gelatin internalization (data not shown) as compared with fibroblasts from normal lungs. IPF MFs were next treated with a variety of agents we have previously reported to elicit rapid MF dedifferentiation, including prostaglandin E_2_ (PGE_2_), the direct adenylyl cyclase activator forskolin, the mitogen fibroblast growth factor-2 (FGF2), and the proteasome inhibitor bortezomib (15, 16, 21). To varying degrees, each of these agents resulted in a significant increase in gelatin internalization capacity after 3 hours (Figure 2D), as determined by initial time course studies (data not shown). By contrast, current FDA-approved therapies for IPF, pirfenidone and nintedanib, failed to increase gelatin internalization in IPF MFs, with the latter significantly reducing it (Figure 2D). Of note, we have previously demonstrated that neither of these approved agents is capable of promoting MF dedifferentiation (18). Confocal microscopy imaging demonstrated that dedifferentiating agents indeed increased the total number of gelatin^+^ puncta as well as of those colocalized with the early endosomal marker EEA1 (Figure 2E), confirming cellular internalization. As observed for fibrotic IPF-derived MFs (Figure 2, A and D), MFs established by TGF-β treatment of normal human lung fibroblasts (15, 16, 38, 39) also demonstrated decreased capacity to internalize gelatin (Figure 2F) that was significantly enhanced by treatment with dedifferentiating agents (Figure 2G).

As previously noted, MFs dedifferentiated in vitro share phenotypic features (e.g., gene expression and apoptosis susceptibility) with mesenchymal cells present during models of fibrosis resolution in vivo. To assess fibroblast gelatin internalization capacity throughout the dynamic course of bleomycin fibrosis, we isolated cells at peak fibrosis (day 21), mid-resolution (day 42), and near-complete resolution (day 63) time points, as previously reported (18). Mouse lung fibroblasts isolated at peak fibrosis demonstrated decreased gelatin internalization capacity as compared with those from saline-treated mice (Figure 2H). Gelatin internalization in fibroblasts was significantly recovered by day 42 and day 63 (Figure 2H). Thus, fibroblasts from fibrotic human and murine lung demonstrate a significant defect in collagen internalization capabilities, and this defect is ameliorated by in vitro treatment with dedifferentiating agents and during spontaneous resolution in vivo.

### MRC2 is reduced in IPF MFs and tissues from human and bleomycin-injured murine lungs.

**Figure 3:**
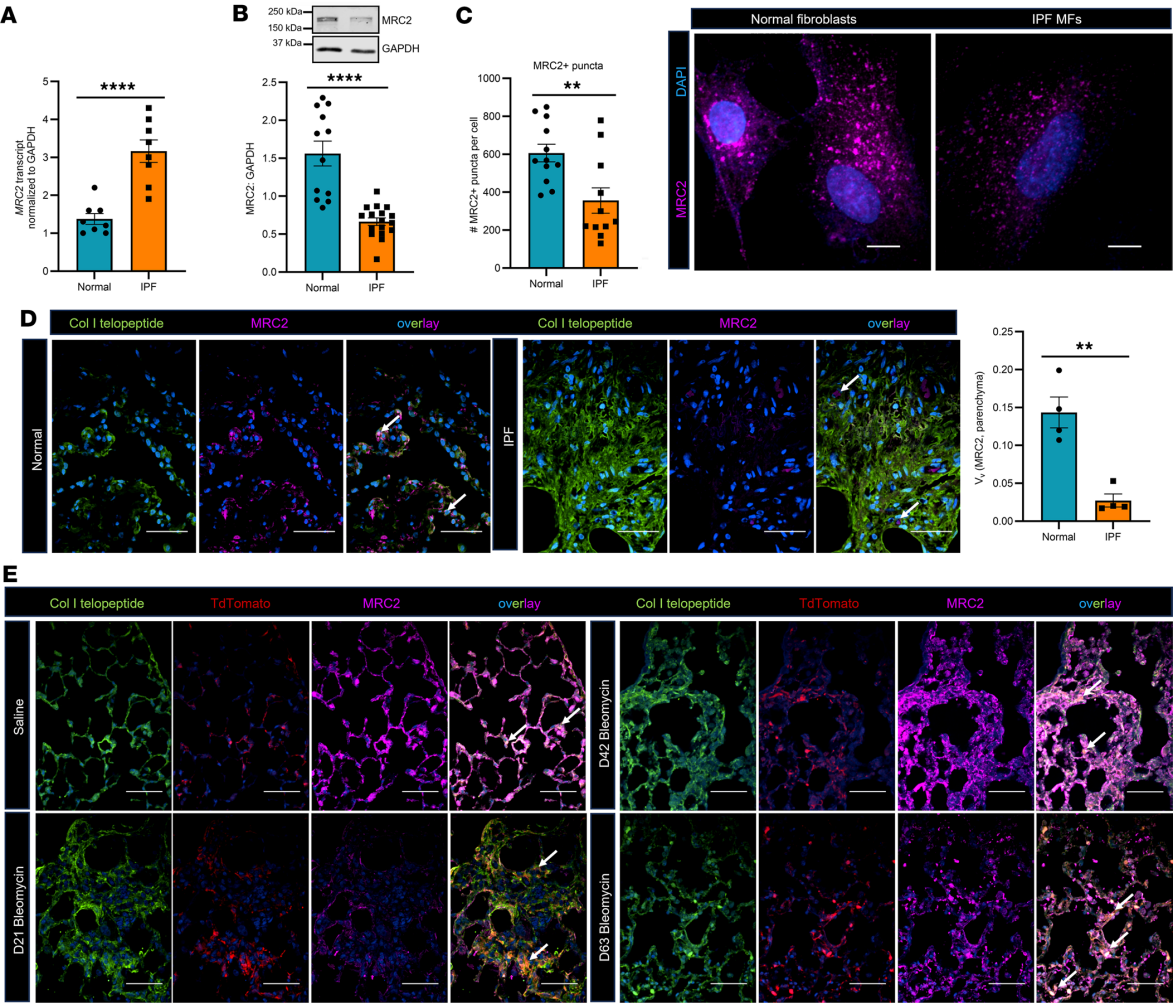
MRC2 expression is decreased in IPF MFs and in human IPF and bleomycin-injured murine lungs. (**A**) qPCR and (**B**) immunoblot analysis of basal MRC2 expression in normal fibroblasts and IPF MFs. (**C**) MRC2 puncta quantification of normal fibroblasts and IPF MFs from confocal images (×100) utilizing CellProfiler. Scale bars: 10 μm. (**D**) Representative images of MRC2 and collagen I telopeptide staining on normal (left panel) and IPF (right panel) lung tissues with quantified volumetric ratio of MRC2 to total lung parenchyma from individual patients’ tissue samples. (**E**) Representative immunofluorescence images of saline-treated and bleomycin-injured lung tissues at peak fibrosis (day 21) and resolution (day 42 and day 63) time points. Tissues were stained for collagen I telopeptide, MRC2, and tdTomato (to denote fibroblasts). Images are representative of an *n* of 4 mice for each treatment group. Scale bars: 50 μm (**D** and **E**). Arrows denote coexpressing cells. Significance of results in **A**–**D** was determined by 2-tailed *t* test, and each individual data point is derived from individual patient cell lines or tissues. **P < 0.01, ****P < 0.0001.

Binding and internalization of degraded collagen fragments in fibroblasts has been predominantly attributed to MRC2, a cell surface receptor found within clathrin-coated pits that is internalized and cycles through the endolysosomal system to shuttle its bound cargo for intracellular degradation (40–46). Consistent with this assertion, MRC2-knockout mice exhibit worse bleomycin-induced fibrosis (47), and MRC2 expression was reported to be reduced in fibroblasts from the lungs of aged mice and humans (12). The expression of MRC2 in MFs from IPF patients has not been evaluated. We therefore determined its RNA and protein expression. Unexpectedly, transcript expression of *MRC2* was significantly higher in IPF MFs than normal fibroblasts (Figure 3A), but protein expression by immunoblot analysis was 2.3-fold lower (Figure 3B). Furthermore, cultured IPF MFs exhibited a parallel 58% decrease in the number of MRC2^+^ puncta compared with normal fibroblasts by immunofluorescent staining (Figure 3C). The distribution of MRC2 in fibrotic versus relatively normal regions of human lung tissue in situ was evaluated by costaining for collagen I telopeptide, a marker of mature extracellular collagen (6, 48). Collagen I telopeptide staining was sparse throughout normal lungs as expected, colocalizing with MRC2^+^ cells in the parenchyma. A similar pattern was observed in the relatively normal regions of IPF lung (Supplemental Figure 2B). In contrast, the more abundant collagen I telopeptide–positive fibrotic areas in IPF tissues demonstrated a paucity of MRC2^+^ cells, with histologic density quantitation demonstrating a significant decrease in the volumetric ratio of MRC2 staining to total parenchyma in IPF tissues (Figure 3D). To elucidate changes in fibroblast MRC2 expression during peak fibrosis and its resolution after bleomycin injury in mice, Col1a2Cre^+^tdTomato^+^ lungs were costained for collagen I telopeptide and MRC2. Saline-treated control lungs demonstrated strong MRC2 staining colocalized in tdTomato^+^ fibroblasts, which was decreased or absent in regions with abundant collagen I telopeptide^+^tdTomato^+^ staining at peak fibrosis. MRC2 expression recovered in tdTomato^+^ fibroblasts during fibrosis resolution at day 42 and day 63, even if tdTomato^+^ fibroblasts were still noted to have collagen I telopeptide positivity (Figure 3E).

### MRC2 is required for the increased gelatin internalization that accompanies MF dedifferentiation.

**Figure 4:**
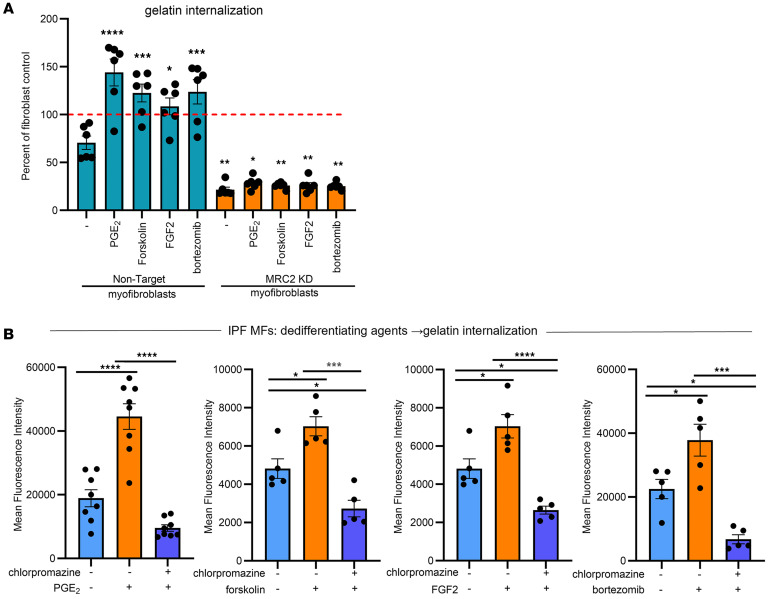
MRC2 is required for dedifferentiating agent enhancement of gelatin internalization. (**A**) TGF-β–elicited MFs that underwent CRISPR-mediated MRC2 silencing were treated with dedifferentiating agents, after which gelatin internalization was assessed by flow cytometry. Significance values determined as compared to non-targeting MF control. No significant differences were found between MRC2-silenced control MFs and those treated with dedifferentiating agents. Red dashed line demarcates fibroblast gelatin internalization, set as control for normalization. (**B**) Gelatin internalization assessed by flow cytometry in IPF MFs after dedifferentiating agent treatment (3 hours) in the presence or absence of the clathrin endocytosis inhibitor chlorpromazine (15 μM). Each data point is derived from an individual patient cell line. Significance for flow cytometric analysis in **A** and **B** was determined by 1-way ANOVA followed by Šidák’s multiple comparisons test (**A**) and Tukey’s multiple comparisons test (**B**). *P < 0.05; **P < 0.01; ***P < 0.001; ****P < 0.0001.

Next, we examined whether MRC2 is required for the increased gelatin internalization associated with MF dedifferentiation. CRISPR-mediated silencing of *MRC2* was performed in MRC5 normal fetal lung fibroblasts (~90% knockdown efficiency, as shown in Supplemental Figure 3A), followed by treatment with TGF-β to establish MFs. TGF-β–differentiated MFs from non-targeting guide RNA–treated control cells demonstrated a 30% reduction in gelatin internalization compared with that of control fibroblasts, consistent with the data in Figure 2F. *MRC2*-knockdown MFs demonstrated a much more substantial reduction in basal gelatin internalization as compared with non-targeting control (Figure 4A), indicating that gelatin internalization is highly dependent on this endocytic receptor. As in Figure 2, D and G, dedifferentiation with PGE_2_, forskolin, FGF2, and bortezomib significantly increased gelatin internalization in non-targeting MFs, but this effect was fully abrogated with *MRC2* silencing (Figure 4A). These data demonstrate the dependence of MFs on MRC2 for both basal and dedifferentiation agent–enhanced gelatin internalization. We next tested the effect of chlorpromazine, a pharmacologic inhibitor of clathrin-coated pit formation, on gelatin internalization. The fact that chlorpromazine abrogated both basal and dedifferentiation-associated increases in gelatin internalization by IPF MFs in a manner similar to *MRC2* silencing further implicates the coordinated MRC2/clathrin pathway in this phenomenon (Figure 4B).

### MRC2 expression is unchanged in IPF MFs upon dedifferentiation.

We next tested whether dedifferentiating agents enhance defective MRC2-mediated gelatin internalization in fibrotic fibroblasts by increasing the deficient expression of MRC2 itself. Surprisingly, immunoblot analysis revealed that none of the effective dedifferentiating agents increased MRC2 protein expression over 1–24 hours of incubation in IPF MFs (Supplemental Figure 3B). Furthermore, despite their decreased gelatin internalization capacity (Figure 2F), TGF-β–elicited MFs demonstrated an increased MRC2 expression compared with control fibroblast lysates (Supplemental Figure 3C), unlike IPF fibroblasts. Finally, neither of the most effective dedifferentiating agents (PGE_2_ and FGF2) increased expression of MRC2 in TGF-β–elicited MFs (Supplemental Figure 3D). Overall, these data suggest that despite a requirement for MRC2 in gelatin internalization, dynamic changes in internalization observed in fibrotic cells either at baseline or with dedifferentiation fail to consistently correlate with alterations in MRC2 expression alone.

### IPF MFs demonstrate reduced endosomal mobilization and lysosomal activity upon gelatin internalization.

**Figure 5:**
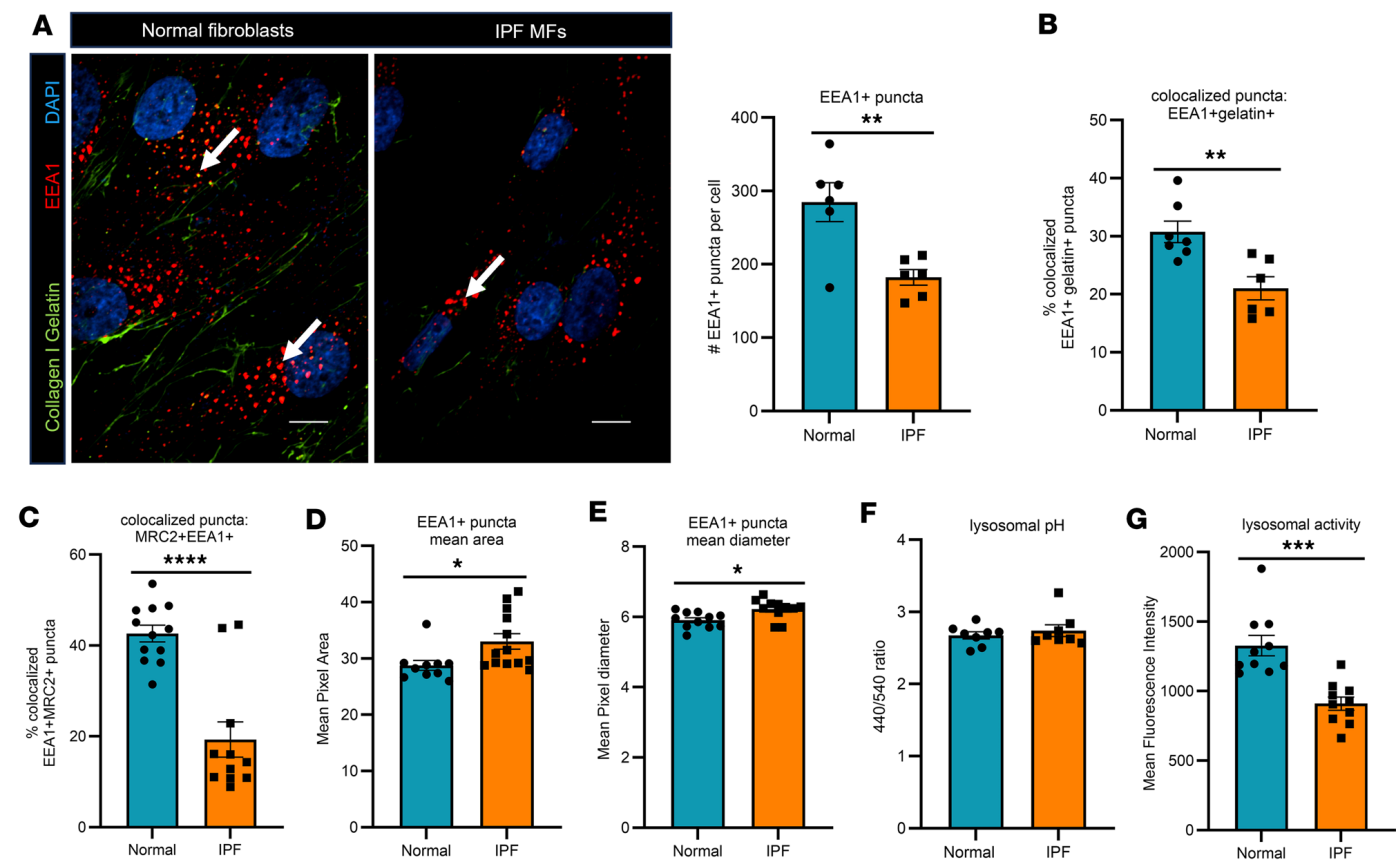
IPF MFs exhibit reduced endosomal vesicles and lysosomal activity. (**A**) Representative confocal images (left) and CellProfiler quantification (right) of total EEA1^+^ vesicles in normal fibroblasts and IPF MFs incubated with fluorescent gelatin. Scale bars: 10 μm. EEA1^+^ puncta colocalization with gelatin (**B**) and MRC2^+^ (**C**) puncta on confocal images of normal fibroblasts and IPF MFs utilizing CellProfiler. EEA1^+^ puncta mean area (**D**) and (**E**) mean diameter measurements from CellProfiler analysis of confocal images of normal and IPF cells. (**F**) Lysosomal pH measured with LysoSensor Yellow/Blue DND-160 in normal fibroblasts and IPF MFs. (**G**) pH-independent lysosomal activity was measured at baseline in normal fibroblasts and IPF MFs after 1-hour treatment with substrate. Each data point in **A**–**G** is derived from an independent patient cell line. Significance of results in **A**–**G** was determined by 2-tailed *t* test. *P < 0.05, **P < 0.01, ***P < 0.001, ****P < 0.0001.

MRC2 and the endolysosomal system as a whole play critical roles in the internalization and intracellular degradation of collagen fragments (49). With MRC2^+^ vesicle numbers found to be decreased in IPF MFs (Figure 3C) but dynamic changes in gelatin uptake not correlating consistently with MRC2 expression, we next set out to determine whether changes in overall endosomal vesicle abundance and/or MRC2 function contributed to reduced gelatin internalization capacity. The quantity of early endosomal vesicles, as marked by EEA1 staining, was significantly decreased in IPF MFs incubated with gelatin as compared with that of normal fibroblasts (Figure 5A). Additionally, a reduced number of EEA1^+^ endosomal vesicles were colocalized with gelatin^+^ puncta (Figure 5B) and MRC2^+^ puncta (Figure 5C) in IPF MFs as compared with normal fibroblasts, demonstrating diminished early endosomal vesicle quantity and function. EEA1^+^ puncta in IPF MFs had a modest but significantly larger mean area (Figure 5D) and mean diameter (Figure 5E).

The protein degradative capabilities of the lysosome are finely tuned, with a pH of 4.5–5.5 enabling optimally efficient cathepsin activation and function (50). IPF MFs did not exhibit a significant difference in lysosomal pH measured with LysoSensor Yellow/Blue DND-160 as compared with normal fibroblasts (Figure 5F). However, when overall lysosomal activity was measured using a pH-independent assay (Figure 5G), MFs from IPF patients demonstrated a 31% decrease as compared with normal fibroblasts, demonstrating a pH-independent mechanism of lysosomal dysfunction.

### Dedifferentiating agents increase endosomal mobilization and lysosomal activity in IPF MFs.

**Figure 6:**
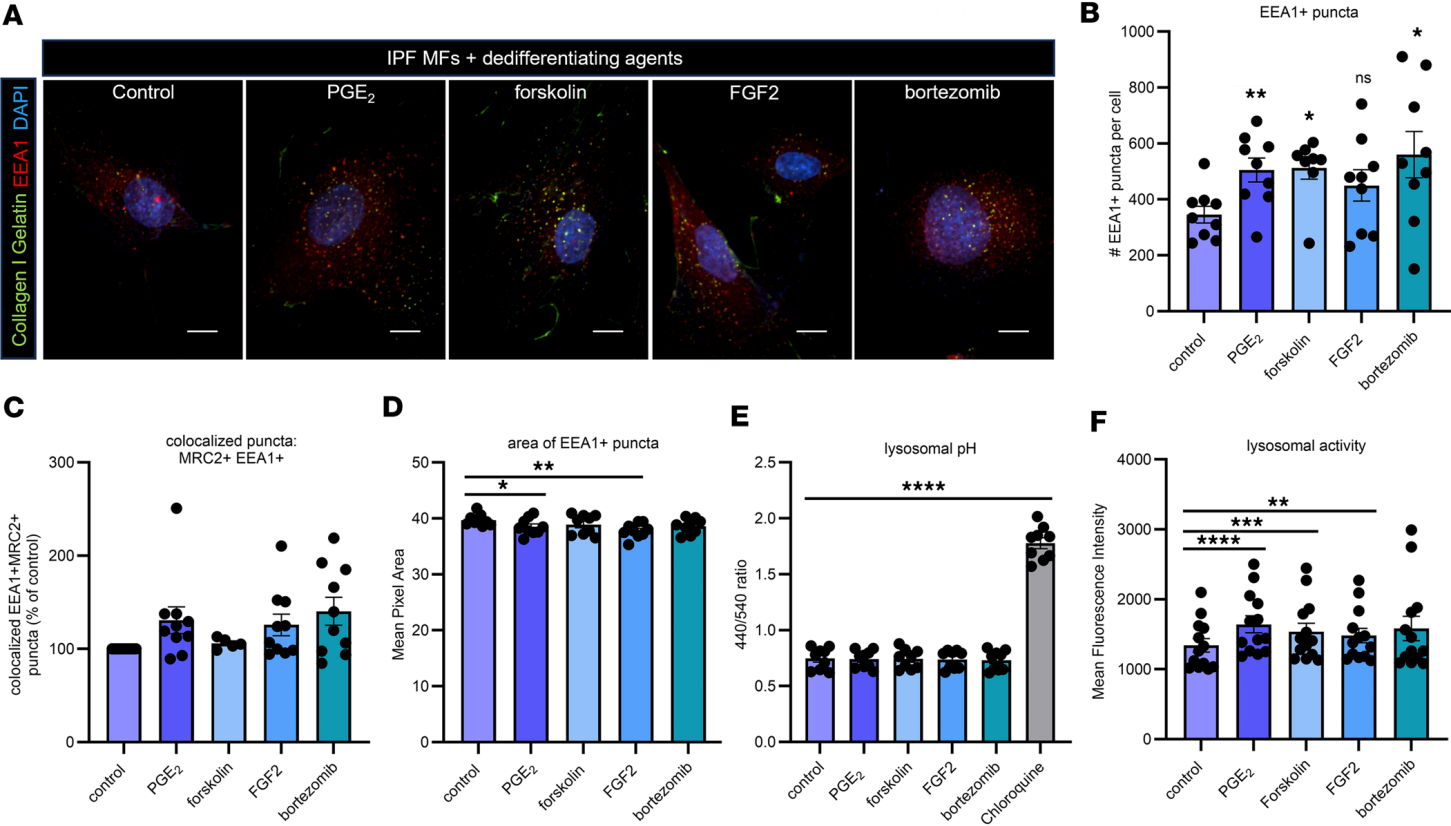
Dedifferentiating agents increase EEA1^+^ vesicle quantity and lysosomal activity in IPF MFs. (**A**) Representative confocal images of EEA1^+^ vesicles in IPF MFs incubated with gelatin after 3-hour treatment with dedifferentiating agents. Scale bars: 10 μm. (**B**) CellProfiler quantification of EEA1^+^ puncta from confocal images after 3-hour dedifferentiating agent treatment of IPF MFs. (**C**) EEA1^+^ colocalization with MRC2^+^ puncta using CellProfiler colocalization analysis on confocal images. (**D**) EEA1^+^ puncta mean area measurements of IPF MFs treated with dedifferentiating agents. (**E**) Lysosomal pH measured with LysoSensor Yellow/Blue DND-160 in IPF MFs after 10 minutes of dedifferentiating agent treatment. Chloroquine (20 μM), an agent known to increase lysosomal pH, was run as a control. (**F**) pH-independent lysosomal activity assessed in IPF MFs after 1-hour dedifferentiating agent treatment. Each data point in **B**–**F** is derived from an independent patient cell line. Significance for **B**–**F** was determined by 1-way ANOVA followed by Dunnett’s multiple comparisons test. *P < 0.05; **P < 0.01; ***P < 0.001; ****P < 0.0001.

Dedifferentiating agents significantly increased gelatin internalization in IPF MFs without increasing levels of MRC2 expression, leading us to explore whether they led to a more efficient mobilization of the endolysosomal machinery. Dedifferentiating agents PGE_2_, forskolin, and bortezomib significantly increased EEA1^+^ vesicle number per cell in IPF MFs, with FGF2 trending towards but not reaching significance (Figure 6, A and B). No significant increase in MRC2 colocalization with EEA1^+^ vesicles (Figure 6C) or total number of MRC2^+^ puncta (data not shown) was found in response to dedifferentiating agents, despite the significant increase in gelatin internalization. This suggests that increases in endosomal vesicle number and/or activity could mediate the enhanced MF gelatin internalization capability in response to dedifferentiating agents. Treatment of IPF MFs with PGE_2_ and FGF2 resulted in modest but significantly decreased EEA1^+^ vesicle area (Figure 6D), suggesting reversal of the enlarged vesicle phenotype noted in IPF MFs in Figure 5D.

G protein–coupled receptor–mediated cAMP signaling has been reported to lower lysosomal pH in a variety of cell types, including fibroblasts (51–54). Treatment with PGE_2_ and forskolin, which signal via cAMP, and FGF2, which does not, significantly increased lysosomal activity in IPF MFs, albeit without impacting lysosomal pH (Figure 6, E and F). Bortezomib failed to significantly increase lysosomal activity (Figure 6F) but resulted in the greatest increase of EEA1^+^ vesicles among the dedifferentiating agents (Figure 6B), suggesting endosomal vesicle formation and/or recycling as the mechanism by which it may increase gelatin internalization. Taken together, these data suggest that the ability of dedifferentiating agents to enhance gelatin uptake in IPF MFs is associated with a concomitant ability to significantly remobilize otherwise impaired endosomal and lysosomal machinery.

## Discussion

ECM accumulates pathologically and impairs lung function in progressive fibrotic disorders such as IPF, yet its clearance is essential if established fibrosis is to be therapeutically targeted to promote resolution (6, 11, 17, 55, 56). A significant gap in knowledge has been our limited understanding of collagen internalization by fibroblasts during both fibrosis and resolution. In this work, we demonstrated that fibrotic human and murine MFs have a reduced capacity to internalize degraded collagen, which is increased during in vivo resolution and upon in vitro treatment with agents that elicit phenotypic dedifferentiation. We identified two cellular defects that contribute to decreased internalization of degraded collagen in IPF MFs, namely (a) reduced MRC2 expression, and (b) decreased endolysosomal activity. Dedifferentiating agents significantly remobilized early endosomal and lysosomal activity, which correlated with increased collagen internalization.

Degradation and clearance of mature ECM collagen in fibrotic lungs has long been attributed to the actions of macrophages. Their role in MMP-dependent collagen degradation is supported by an extensive body of literature (11, 57–59). Likewise, their participation in collagen internalization via mannose receptors has been reported in vitro (60, 61), while another study identified an in vivo role for the secreted glycoprotein Mfge8 that binds collagen via its discoidin domain for macrophage-mediated removal (9). While macrophage depletion during the late (i.e., resolution) phase of bleomycin fibrosis was found to impede spontaneous resolution (62), it is not known whether this relates to the absence of their participation in collagen degradation and/or clearance.

The role of fibroblasts in collagen clearance has received comparatively less attention. Moreover, there is a paucity of information regarding these processes in fibrotic lung disorders, including IPF, and virtually nothing is known about them in the context of spontaneous fibrosis resolution. Our in situ use of CHP to stain for collagen degradation products revealed localized distribution surrounding fibroblasts that was increased in IPF (Figure 1, A and C). In the bleomycin mouse model, CHP staining surrounding fibroblasts was similarly increased at peak fibrosis yet declined during spontaneous resolution (Figure 1D). These in situ data parallel another study demonstrating increased CHP in IPF tissues (8), in addition to a number of clinical studies reporting that plasma levels of both MMPs as well as various collagen degradation products serve as biomarkers for IPF and correlate with disease progression (3–5, 29, 30). This apparent paradox of increased collagen degradation in the setting of disease progression highlights the importance of understanding mechanisms governing cellular internalization and clearance of collagens in fibrotic tissue. Specifically, how collagen internalization is altered in fibroblasts and how this might contribute to dynamic changes in ECM imbalance during fibrosis and its resolution was the primary focus of the current investigation.

Despite multiple reports indicating that loss of MRC2-dependent internalization of degraded collagen by fibroblasts contributes to fibrosis (12, 42, 47, 60, 63, 64), we are aware of no studies that have explored this process in IPF patient–derived lung MFs. Information is similarly lacking about MRC2 function during fibrosis resolution, and whether lung MF dedifferentiation influences MRC2 expression and function. Our current findings link resolution of lung fibrosis with an increase in fibroblast-mediated collagen internalization. These data are consistent with a recent report of impaired resolution from bleomycin-induced fibrosis in aged mice in association with reduced MRC2 expression in lung fibroblasts (12). An additional recent report linked impaired bleomycin fibrosis resolution in response to treatment with a BCL-2 inhibitor with concomitant ER-stress-driven downregulation of MRC2 (65). The requirement of MRC2-mediated collagen internalization for fibrosis resolution bears direct investigation in future studies.

Recent single-cell sequencing studies of IPF and normal human lungs have demonstrated modest increases in *MRC2* expression in IPF-derived fibroblast populations (66–70). Our studies confirm an increase in *MRC2* at the transcript level but found that protein expression of MRC2 was reduced. Specifically, we found that MRC2 protein expression was decreased in IPF MFs in culture, in fibroblasts from stained lung sections of human IPF, and in fibroblasts within bleomycin-injured mouse lungs. This is concordant with a past report demonstrating decreased lung MRC2 expression 14 and 21 days after bleomycin challenge (47). However, this pattern differs from studies in both liver (64) and renal (63) fibrosis, in which MRC2 expression was increased in fibrotic regions. These disparate findings suggest cell- and tissue-specific differences in how MRC2 is regulated and how it influences ECM homeostasis.

In addition to reduced MRC2 expression in IPF MFs, we also found a constellation of endolysosomal abnormalities therein, including decreased endosomal vesicle numbers, increased vesicle size, and decreased lysosomal activity in IPF MFs. To our knowledge, such abnormalities have not been reported in fibrotic lung disease. However, a generalized increase in endosomal vesicle size has been reported in the brains of Alzheimer disease patients and in the brain tissues and skin fibroblasts of patients with Down syndrome (71). While pulmonary fibrosis occurs in many individuals with Hermansky-Pudlak syndrome, a rare genetic disease characterized by dysregulated biogenesis and function of lysosome-related organelles (72), the specific role of fibroblast lysosomal-related defects in this and other lysosomal storage diseases remains to be determined.

Phenotypic dedifferentiation of MFs increased their capacity to internalize degraded collagen and enhanced their endosomal and lysosomal activity, a phenomenon not previously reported to our knowledge. Lysosomes in skin fibroblasts from patients with Alzheimer disease have been reported to exhibit increased pH, with cAMP treatment reducing pH levels and improving lysosomal activity (51). Additionally, a study utilizing dopamine receptor agonism to elicit cAMP generation in TGF-β–elicited lung MFs observed a significant decrease in lysosomal pH that coincided with increased fibrillar collagen internalization and lysosomal degradation (52). Our findings demonstrate a pH-independent lysosomal abnormality associated with defective gelatin uptake in fibrotic lung fibroblasts (Figure 5). The mechanism underlying this endolysosomal defect remains to be defined. Nevertheless, these data suggest the presence of multiple mechanisms of endolysosomal dysfunction in fibrotic mesenchymal cells. As efficient extracellular clearance and intracellular degradation of mature collagen is crucial for fibrosis resolution, a better understanding of how lung fibroblasts/MFs contribute to this process will be important to develop future therapies for IPF.

Collagen homeostasis in tissues requires a balance among its synthesis, extracellular degradation, internalization, and intracellular degradation (6, 56, 73). A recent study demonstrated SEL1L-mediated pro-collagen binding and sensing during synthesis that was necessary for positively regulating *MRC2* transcript expression (74). While the exact mechanism by which SEL1L promoted *MRC2* expression was not elucidated, this study highlighted the finely tuned regulation that occurs between collagen synthesis and internalization. While we are aware of no studies demonstrating control of MRC2 and collagen internalization by the extracellular degradation machinery, there is a report demonstrating that MRC2 acts to limit MMP14 membrane localization and subsequent MMP2 activation that is dependent on its collagen binding capacity (75). Additional nodes of the collagen regulatory pathway that may impact degraded collagen internalization include the intracellular turnover of collagen prior to secretion (76), in addition to endocytic recycling and fibrillogenesis signaling programs (77–79). From this body of work, it is evident that substantially more research is needed to fully understand the complex interplay among these components of the collagen cycle.

It is notable that cAMP-independent dedifferentiating agents FGF2 and bortezomib shared with the cAMP-dependent agents PGE_2_ and forskolin the ability to enhance gelatin uptake in association with increased lysosomal activity and/or EEA1^+^ puncta numbers. We currently do not know whether the enhanced uptake promoted by these diverse agents reflects the fact that uptake itself can be modulated by multiple intracellular signaling pathways, or whether enhanced uptake is instead the conserved property of the dedifferentiated MF, regardless of how this was achieved. However, the fact that a greater capacity for gelatin uptake was also observed in lung fibroblasts isolated during the process of spontaneous resolution from experimental fibrosis prompts us to favor the latter possibility. Better understanding of this important process is a key goal for future research.

It is appropriate to consider whether the endolysosomal defects demonstrated in our study may be associated with defects in autophagy, which have been identified in various cell types including fibroblasts and implicated in IPF pathogenesis (80-85). Indeed, there is overlap in the regulation of the endosomal and autophagosome machinery (86). Determining whether MRC2 loss in IPF tissues and MFs is a result of defective endosomal machinery, the result of dysregulated autophagy signaling, or an independent phenomenon, warrants further investigation. Future studies will be necessary to assess whether reduced EEA1^+^ and MRC2^+^ vesicle numbers and lysosomal activity in IPF MFs contribute to disease pathogenesis independently or as the result of defective autophagy responses, and whether restoration of lysosomal activity is necessary for MF dedifferentiation.

A limitation of our study is that we did not address the possible role of specific subsets of fibroblasts in collagen internalization. Future studies should address this issue in the context of both fibrosis development and its resolution. Although we focused in this study on the relatively understudied role of fibroblasts in collagen internalization, we acknowledge the role of macrophages and the fact that the relative contribution of these two cell types to degradation and clearance of collagen remains to be fully defined, especially in vivo. Furthermore, crosstalk between macrophages and fibroblasts in carrying out these critical functions and particularly in the context of fibrosis resolution is of importance and deserves future investigation.

In summary, we have identified an impaired ability of MFs from patients with IPF and fibrotic mouse lung to internalize collagen fragments, owing to defects in both MRC2 expression and endolysosomal function. These functional and mechanistic defects are mitigated during fibrosis resolution and can be reversed upon treatment with dedifferentiating agents. These results highlight the importance of fibroblasts as dynamic participants in the ECM clearance essential for fibrosis resolution and suggest novel therapeutic approaches that may have transformative potential.

## Methods

### Sex as a biological variable.

As pulmonary fibrosis occurs in both sexes, we utilized male and female animals in murine studies, with no difference in findings noted by sex. All data presented include results from both sexes.

### Cell culture.

Normal adult– and IPF-derived fibroblast lines and fixed tissues were obtained after patients provided written, informed consent. Fibroblasts and tissues were isolated from lung explants of IPF patients undergoing lung transplantation and lung tissue from deceased donors without fibrosis was provided by Gift of Life, Michigan. Fibroblasts were isolated and cultured as previously described (87). Briefly, lung tissues were minced and enzymatically digested to a single-cell suspension in a solution containing 1 mg/mL collagenase A (Roche, 10103578001), 2–4 U/mL elastase (Worthington, LS002274), and 0.1 mg/mL DNase (Roche, 10104159001) in DMEM for 45–60 minutes at 37°C. EpCAM^+^, CD31^+^, and CD45^+^ populations were then depleted utilizing biotin-conjugated antibodies (Invitrogen: EpCAM clone 1B7, 13-9326-82; CD45 clone HI30, 13-0459-82; CD31 clone WM59, 13-0319-82) and streptavidin-conjugated magnetic beads, following the manufacturer’s protocol (Promega, Z5481). The resulting cell suspension was then cultured on tissue culture plastic and expanded by serial passaging. Validation of fibroblast cultures was performed by flow cytometry to ensure no contaminating EPCAM^+^, CD31^+^, or CD45^+^ cells were present, as well as qPCR to ensure E-cadherin (*CDH1*) negativity and *COL1A1* expression. Sex and age distribution of the patients from whom fibroblasts were derived is outlined in Supplemental Table 1, with no statistically significant difference in sex or age distribution noted between normal and IPF individuals, as determined by Fisher’s exact test. MRC5 normal fetal lung fibroblasts were obtained from the American Type Culture Collection. All cells were cultured in low-glucose DMEM (Invitrogen) supplemented with 10% FBS (Biowest), 100 U/mL penicillin, and 100 μg/mL streptomycin (both from Invitrogen) prior to plating. Once plated, cells were serum starved overnight and treated with dedifferentiating agents (IPF MFs) or MFs were established (when using MRC5 line) first by treatment with TGF-β (R&D Systems) for 48 hours prior to dedifferentiation. In vitro data were derived from a minimum of 3 individual experiments, each from a unique patient cell line, and the results are presented as mean ± SEM.

### Reagents.

Recombinant human TGF-β (used at a concentration of 2 ng/mL; 7754-BH) and FGF2 (50 ng/mL; 233-FB) were purchased from R&D Systems and resuspended in filter-sterilized 4 mM HCl plus 0.1% BSA and PBS, respectively. PGE_2_ (500 nM; 14010), the direct adenylyl cyclase activator forskolin (20 μM; 11018), and antifibrotic drugs pirfenidone (1 mM; 13986) and nintedanib (2 μM; 11022) were purchased from Cayman Chemicals. The 20S proteasome inhibitor bortezomib (10 nM; 5.04314), chloroquine (20 μM; C6628) and clathrin endocytosis inhibitor chlorpromazine (15 μM; C8138) were purchased from MilliporeSigma. Oregon Green 488–conjugated gelatin from pig skin (5 μg/mL; G13186) and Fast SYBR Green Master Mix (4385612) were purchased from Thermo Fisher Scientific.

### Gelatin internalization assay.

Fibroblasts were plated into 6-well plates to obtain 80% confluence the following day, followed by overnight serum starvation. IPF MFs were treated with dedifferentiating agents for 2 hours or basal internalization was assessed utilizing 5 μg/mL of Oregon Green 488–conjugated gelatin added directly to each well for 1 hour at 37°C. Cells were then washed with PBS, quenched with 0.05% trypan blue in PBS (to quench bound extracellular and uninternalized collagen signal), and washed prior to trypsinization and resuspension for FACS analysis. Gelatin internalization was assessed utilizing a BD LSRFortessa or Bio-Rad Ze5 flow cytometry platform.

### Lysosomal activity assay and lysosomal pH determination.

Lysosomal pH was determined using LysoSensor Yellow/Blue DND-160 (Thermo Fisher Scientific, L7545). Briefly, 10,000 fibroblasts were plated per well into black, clear-bottom 96-well plates and allowed to adhere overnight. Cells were then incubated with 5 μM lysosensor substrate diluted in DMEM for 1 minute, the reagent removed, and cells washed with PBS. Dedifferentiating agents were then added to appropriate wells in PBS, or basal lysosomal pH readings were assessed by measuring fluorescence on a microplate reader over a time interval between 1 and 30 minutes. The 10-minute time point was chosen for final data graphs, as optimal signal intensity was found at that time. Data are presented as the ratio of emissions (440 nm/540 nm). Lysosomal activity was determined utilizing a proprietary pH-insensitive self-quenched substrate that is internalized by endocytic transport and cleaved by lysosomal proteases, resulting in a strong fluorescence signal proportional to intracellular lysosomal protease activity (Abcam, ab234622). Fibroblasts were plated into 6-cm culture plates and adhered overnight. Cultures were synchronized for 3 hours with DMEM plus 0.5% FBS and 5 μg/mL substrate added for 1 hour in the presence or absence of dedifferentiating agents. Cells were subsequently trypsinized, washed with 1× lysosomal buffer, and resuspended in PBS for FACS analysis on a Bio-Rad Ze5 cytometer.

### Animal studies.

WT male and female C57BL/6 mice were obtained from The Jackson Laboratory and used at 8–10 weeks of age. tdTomato reporter strain B6.Cg-Gt(ROSA)26Sor^tm14(CAG-tdTomato)Hze^/J (The Jackson Laboratory, 007914) was crossed with *Col1a2*^CreERT2+/0^ mice (The Jackson Laboratory, 029567) to generate tamoxifen-inducible fibroblast-specific tdTomato-labeled mice. Genotyping was performed on tail-extracted DNA as previously described (18).

Pulmonary fibrosis was induced utilizing single-dose oropharyngeal bleomycin administration (1.0 U/kg; Meitheal, NDC 71288-106-10), as described previously (18, 20). Labeling of *Col1a2*^CreERT2+/0:^B6.Cg-Gt(ROSA)26Sor^tm14(CAG-tdTomato)Hze^/J fibroblasts during fibrogenesis was performed utilizing tamoxifen chow (40 mg/mouse/day; Inotiv, TD.130859) administration from days 9 to 21 after bleomycin administration. Mice were sacrificed on days 21, 42, or 63 and lungs perfused with cold PBS followed by formalin fixation and paraffin embedding to obtain tissue sections or collagenase digestion to isolate fibroblasts. Representative histological images for each time point after Masson’s trichrome staining are included in Supplemental Figure 2A. These data confirm our previously reported time course for fibrosis resolution after bleomycin administration (18). For fibroblast isolation, lungs were perfused, minced, and placed into a 0.1% collagenase A solution and rotated for 30 minutes at 37°C. Tissue bits were then dissociated further using an 18G needle attached to a 5 mL syringe and this single cell suspension passed through a 50 μm cell strainer. Cells were then pelleted at 500*g* for 10 minutes, resuspended in MACS buffer, and CD45^+^ cells depleted using anti-CD45 magnet-conjugated beads (Miltenyi Biotec, 130-052-301), following the manufacturer’s protocol. CD45^–^ cells were then plated in serum-containing DMEM to allow for fibroblast expansion. Fibroblasts were passaged 3 times to eliminate contaminating cells and identification verified by flow cytometric analysis before use in cell-based assays. Mouse lung fibroblasts at passage 3 were found to be 96.8% ± 0.75% PDGFRα^+^, 1.8% ± 0.77% CD45^+^, 0.24% ± 0.13% EpCAM^+^, and 0.01% ± 0.01% CD31^+^, indicative of a substantially pure fibroblast culture.

### CRISPR-mediated MRC2 silencing.

An inducible fibroblast line capable of CRISPR/Cas9-mediated deletion of *MRC2* was generated by infection of MRC5 fibroblasts with lentiviral particles containing pLentiCRISPR v2 TLCV2-delete GFP plasmid (Addgene, 133302) with the following gRNA sequence targeting exon 12 inserted: *MRC2*: 5′-GGGCCTCGGACACCAAACTC-3′. A non-targeting sequence (5′-AAATGTGAGATCAGAGTAAT-3′) (Thermo Fisher Scientific, A35526) inserted into TLCV2-deleteGFP served as a negative control. Puromycin selection (5 μg/mL) for 48 hours was initially performed to select for fibroblasts containing the vector and conditional *MRC2* deletion was achieved by addition of doxycycline (1 μg/mL; Cayman Chemicals) to conditioned medium.

### Western blot.

Cells were lysed with RIPA buffer supplemented with protease inhibitors (Roche Diagnostics, 11836153001) and a phosphatase inhibitor cocktail (EMD Biosciences, 524624 and 524625) and spun to clarify debris. Proteins were separated by SDS-PAGE and transferred to PVDF membranes followed by blocking with 5% BSA. Membranes were probed with goat anti-human MRC2 (R&D Systems, AF5770) and mouse anti-human GAPDH (Thermo Fisher Scientific, MA5-15738) with IRDye 800CW (Licor) secondary antibody used for detection on a Licor Odyssey M digital imager.

### qPCR.

Analysis of *MRC2* expression was performed on total cellular RNA after isolation utilizing a spin RNeasy kit (Qiagen). cDNA was prepared with High-Capacity cDNA Reverse Transcription Kit (Applied Biosystems), and amplification performed using Fast SYBR Green Master Mix on a StepOne real time PCR system (Applied Biosystems). Relative expression of *MRC2* transcripts was determined utilizing the 2^–ΔΔCt^ method after normalizing to levels of the housekeeping gene *GAPDH*. Primer pair sequences used for qPCR of human *MRC2* were forward (5′-CACTGCTATTCTTTCCACAT-3′) and reverse (5′-ACATTCTCCATCTCATCCA-3′).

### Cell and tissue immunofluorescent staining and confocal microscopy.

IPF MFs or normal fibroblasts were plated into 4-well chambered cell culture slides (Falcon, 354114), allowed to adhere overnight, and serum starved for 16 hours. Following serum starvation, cells were treated with gelatin for internalization assays, with or without dedifferentiating agents, then washed twice with chilled PBS, fixed with 4% formaldehyde for 10 minutes, then washed again with PBS to remove residual fixative. Blocking and permeabilization were achieved by incubating the slides for 1 hour in PBS containing 2% BSA plus 0.1% Triton X-100 (Sigma-Aldrich). Cells were then incubated with either anti-human MRC2 (1:100; R&D Systems, AF5770) or anti-human EEA1 (1:100; Abcam, ab2900) overnight at 4°C. The following day, chambers were washed with PBS, incubated with secondary antibodies for 1 hour, then washed again and mounted with a coverslip utilizing ProLong Diamond antifade mountant containing DAPI (Invitrogen) to visualize nuclei.

Immunohistochemical staining on 5-μm-thick human and mouse formalin-fixed, paraffin-embedded sections was performed utilizing standard lab procedures for clearing tissues; antigen retrieval was not employed to avoid collagen degradation that might skew the CHP staining signal that was intended to capture degraded collagen only. Tissues were then blocked for 1 hour with 2% horse serum, followed by avidin biotin blocking steps following manufacturer’s protocol (Vector Labs, SP-2001). Biotin-conjugated CHP (3Helix, BIO60) was prepared to 10 μM final concentration following the manufacturer’s protocol and applied to slides overnight at 4°C. The following day, after 2 PBS washes, tissues were incubated with ready to use ABC reagent (Vector Labs, PK-7100) and developed utilizing Cy5 Tyramide (Akoya Biosciences, SAT705A001EA) according to the manufacturer’s protocol. Slides were then retrieved in citrate buffer for 6 minutes, and blocked and incubated with either rabbit anti-CD206 (1:100; Abcam, ab64693) or rabbit anti-human PDGFR-α (1:100; LS-BIO, LS-B2683) overnight at 4°C. For mouse sections, anti-CD68 (1:100; Abcam, ab125212) and anti-tdTomato (1:200; Rockland, 600-401-379) primary antibodies were utilized. Col1agen I telopeptide (1:100; Thermo Fisher Scientific/Invitrogen, PA5-35380) and MRC2 (1:100; R&D Systems, AF5770) were utilized for mouse and human slides after retrieval as described above. Mouse and human tissues were imaged utilizing a 40× water objective and cell imaging was performed using a 100× oil objective on a Nikon X1 Yokogawa spinning disk confocal microscope.

### Histomorphometry analyses.

Measurements of the density of MRC2 or degraded collagen were performed on 3–6 non-overlapping images taken from slides fluorescently costained for MRC2 and collagen I telopeptide, or CHP and CD206 (to quantify in macrophage containing areas) or PDGFRα (to quantify in fibroblast containing areas of the lung). Histomorphometry analyses were done according to ATS/ERS standards for Quantitative Assessment of Lung Structure (88), with degraded collagen density measured as the area containing CHP fluorescence signal within macrophage- or fibroblast-rich regions of the lung parenchyma. MRC2 density was measured as the relative area containing MRC2 fluorescence signal within lung parenchyma. Morphometric measurements were made by an independent pathologist blinded to study group using ImageJ 1.8.0 (NIH).

### CellProfiler analysis.

CellProfiler version 4.2.8 was downloaded from https://cellprofiler.org/ and used for determination of puncta numbers, colocalization, diameter, and area measurements. A CellProfiler-published speckle counting pipeline (https://cellprofiler.org/examples) with modifications was utilized for puncta quantification and measurements, with colocalization pipeline used to determine gelatin, EEA1, and MRC2 colocalization. Three to 8 images were utilized for each cell line or dedifferentiating agent condition in the pipeline analyses, with mean results presented for each.

### Statistics.

Statistical analysis was performed using GraphPad Prism software version 10.4.1. Data are presented as mean ± SEM and were analyzed for statistical significance by 1-way ANOVA with Šídák’s or Dunnett’s multiple-comparison tests or paired/unpaired 2-tailed *t* test, as appropriate. A *P* value of less than 0.05 was considered significant.

### Study approval.

Normal human adult– and IPF-derived fibroblast lines and fixed tissues were obtained via a protocol (HUM00105694) reviewed and approved by the University of Michigan Institutional Review Board. All animal experiments were approved and carried out in accordance with the University of Michigan IACUC and conformed to the Animal Research: Reporting of In Vivo Experiments (ARRIVE) guidelines.

### Data availability.

Values for all data points in graphs are reported in the Supporting Data Values file.

## Author contributions

The project was conceptualized by MPG and NMW. NMW, SMF, JS, and MPG designed the in vitro and in vivo experiments. Experiments were performed by NMW and JS. Data were analyzed by NMW, SG, TSB, and SMF. Intellectual contributions were provided by SG, TSB, and SKH. Patient-derived normal and IPF fibroblasts were provided by SKH. The manuscript was written by NMW, SMF, and MPG. All authors reviewed the manuscript.

## Conflict of interest

The authors have declared that no conflict of interest exists.

## Funding support

This work is subject to the NIH Public Access Policy, with acceptance of this federal funding giving the NIH the right to make this work publicly available in PubMed Central.

- NIH grants R35 HL144979 (to MPG), K08 HL163178 (to SMF), R01 HL175555 (to TSB), and R01 HL162963 (to SKH).

## Supplementary Material

Supplemental data

Unedited blot and gel images

Supporting data values

## Version 1 05/19/2026

In-Press Preview

## Version 2 07/08/2026

Electronic publication
