# Supplementary material for: MRC2-mediated collagen internalization is reduced in fibrotic lung fibroblasts and increased upon phenotypic dedifferentiation
Source: JCI Insight. 2026 May 19;11(13):e201712. doi: 10.1172/jci.insight.201712 (PMC13460822; doi:10.1172/jci.insight.201712)
Supplement: Supplementary file 1 [file jciinsight-11-201712-s226.pdf]

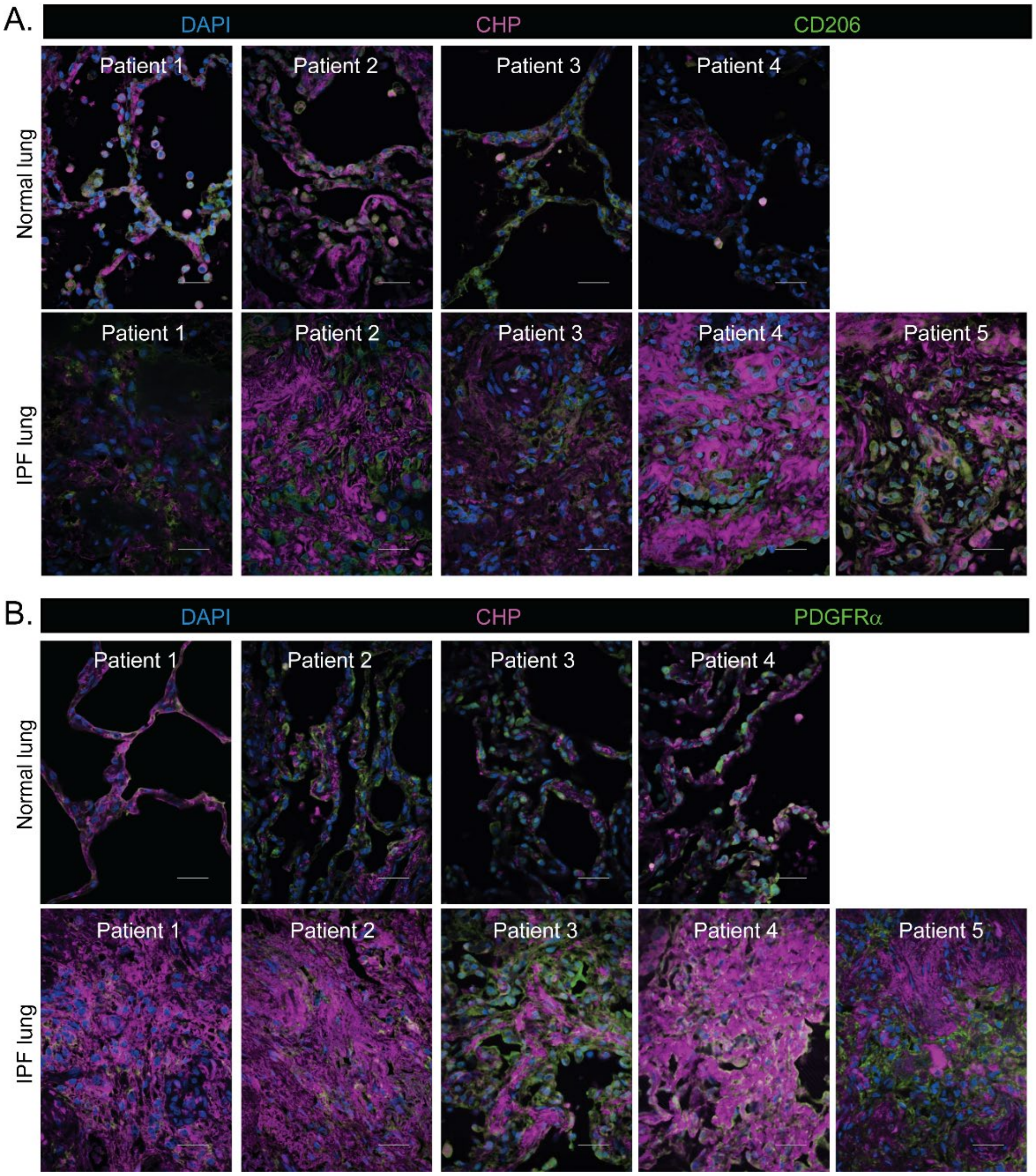

Supplemental Figure 1. Representative images of sections from normal (top) and IPF (bottom) human lungs showing CHP fluorescent co-localization with (**A**) CD206 to label macrophages or (**B**) PDGFR-α to label fibroblasts. Scale bars indicate 50 µm.

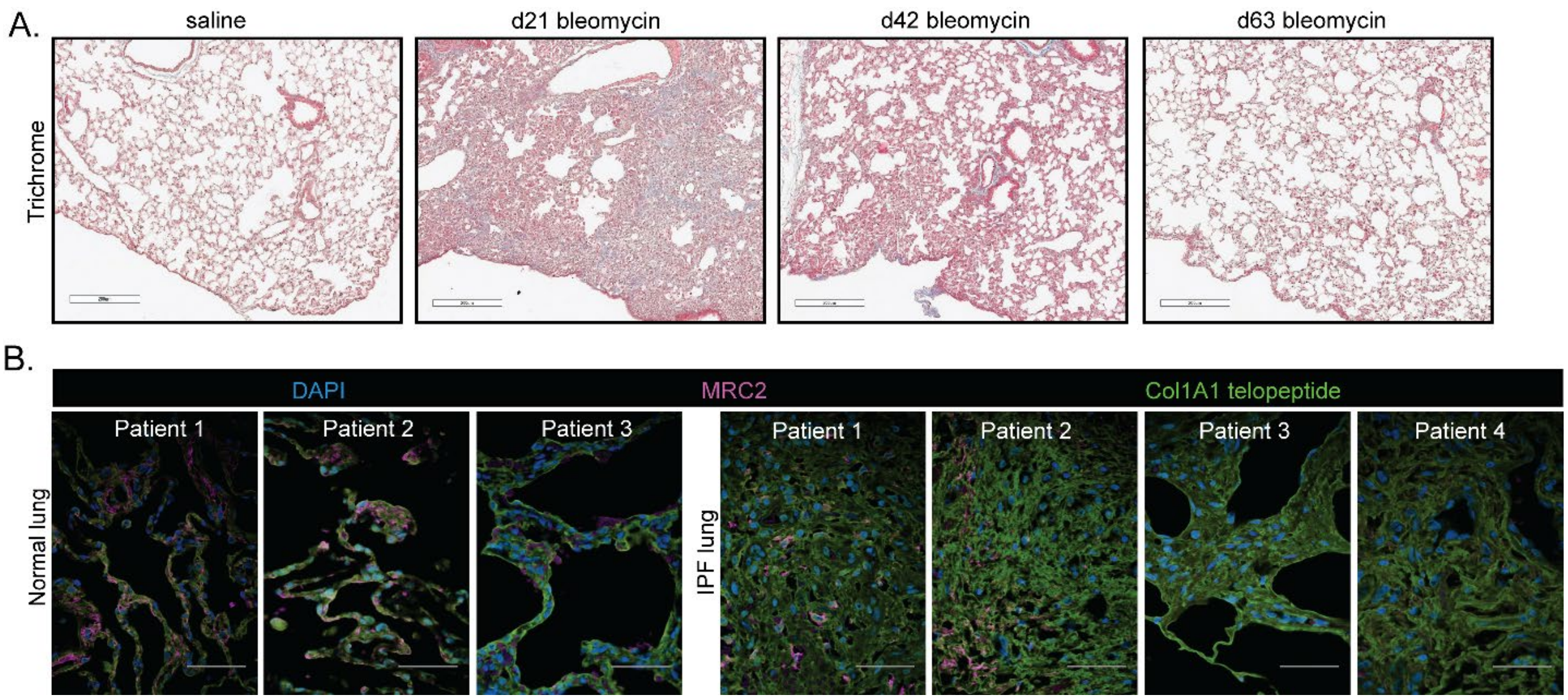

Supplemental Figure 2. (**A**) Representative 10x trichrome images of saline and bleomycin-injured lung tissues at peak fibrosis (d 21), mid-resolution (d 42) and full resolution (d 63) time points. Images are representative of an n of 4 mice for each treatment group. Scale bars represent 200 µm. (**B**) Representative images of sections from normal (left) and IPF (right) human lungs showing MRC2 fluorescence co-staining with collagen I telopeptide indicating fibrillar intact collagen. Scale bars indicate 50 µm.

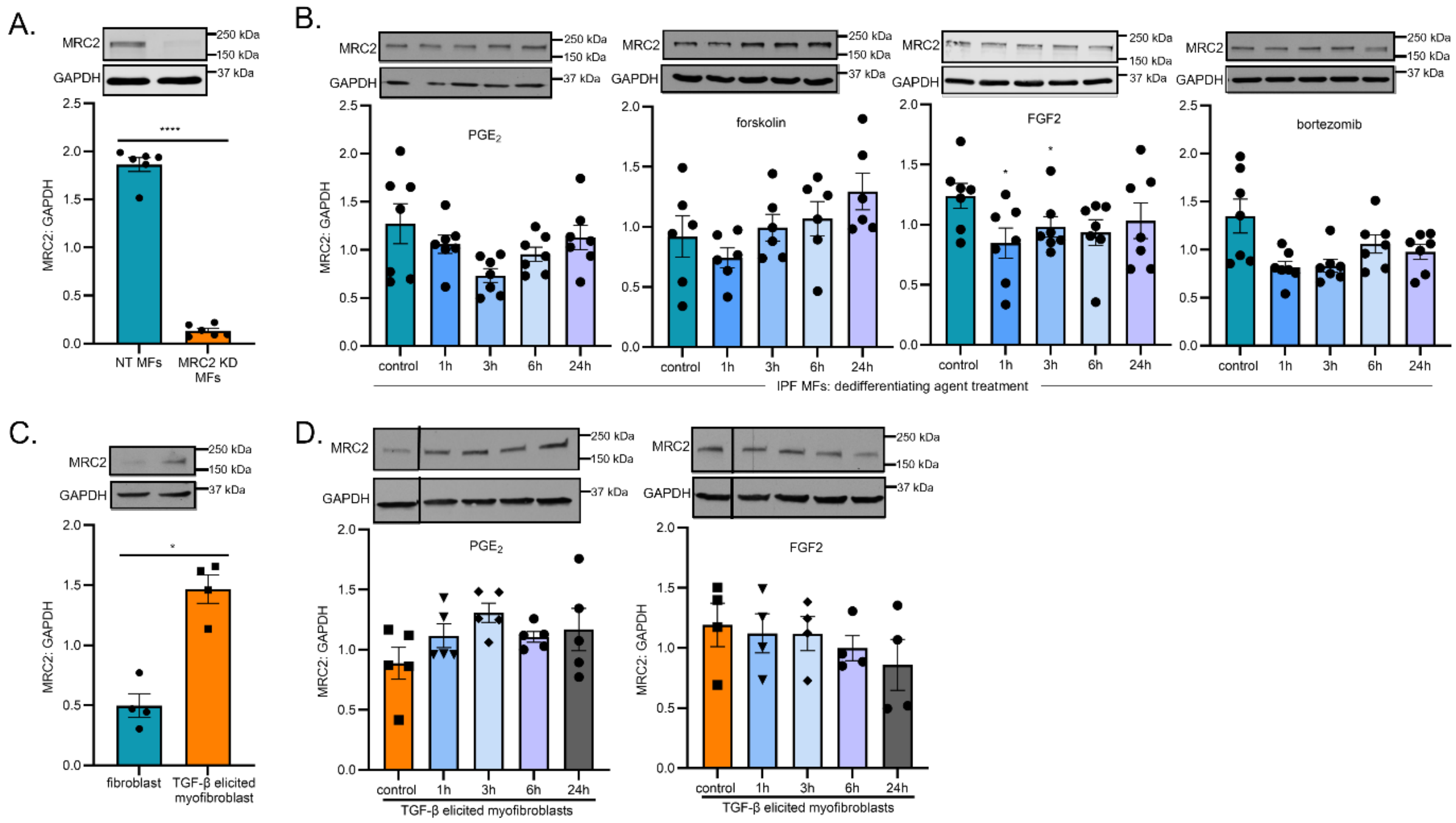

Supplemental Figure 3. (**A**) MRC2 knockdown efficiency by immunoblot analysis in TGF-β-elicited MFs that underwent CRISPR-mediated MRC2 silencing. (**B**) Immunoblot analysis of MRC2 in IPF MFs after 1-24 h of dedifferentiating agent treatment. Each data point is derived from an independent patient cell line. (**C**) MRC2 immunoblot analysis of TGF-β-elicited MFs at baseline and (**D**) after dedifferentiation induced by PGE_2 and FGF2. Immunoblot images in (**D**) were run on the same gel, but were noncontiguous, as designated by the solid black line between lanes. Significance in **A** & **C** was determined by 2-tailed t-test, while that in **B** & **D** was determined by 1-way ANOVA. * p<0.05, **** p<0.0001.

Supplemental Table 1.

Age and Sex Characteristics of Patients from whom Lung Fibroblasts were Obtained			
	**Sex Distribution:**		**Mean Age ± SEM (years):**
Normal lung derived fibroblasts	Male	15 (62.50%)	40.80 ± 4.41
	Female	9 (37.50%)	44.40 ± 4.97
IPF lung derived fibroblasts	Male	18 (81.82%)	57.04 ± 2.40
	Female	4 (18.18%)	58.5 ± 3.38
Fisher's Exact Test:		p=0.1968	p>0.9999
