# Supplementary material for: MRC2-mediated collagen internalization is reduced in fibrotic lung fibroblasts and increased upon phenotypic dedifferentiation
Source: JCI Insight. 2026 May 19;11(13):e201712. doi: 10.1172/jci.insight.201712 (PMC13460822; doi:10.1172/jci.insight.201712)
Supplement: Supplementary file 2 [file jciinsight-11-201712-s227.pdf]

Figure 3B. Uncropped/unedited blot images

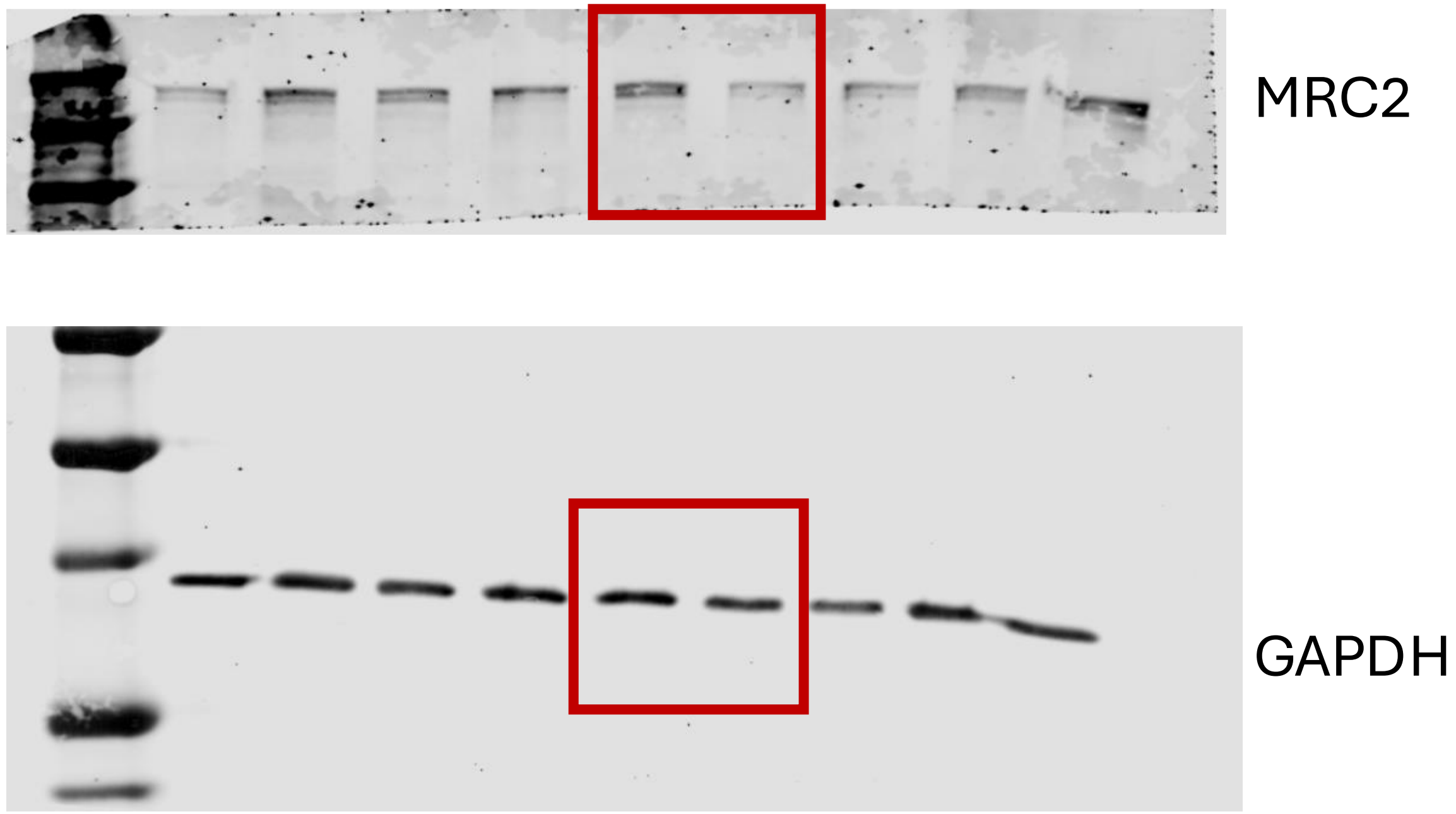

Supplemental Figure 3A. Uncropped/unedited blot images

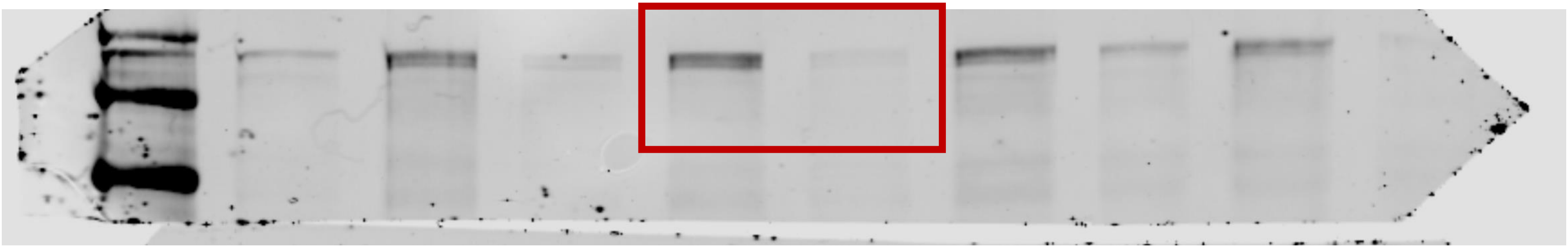

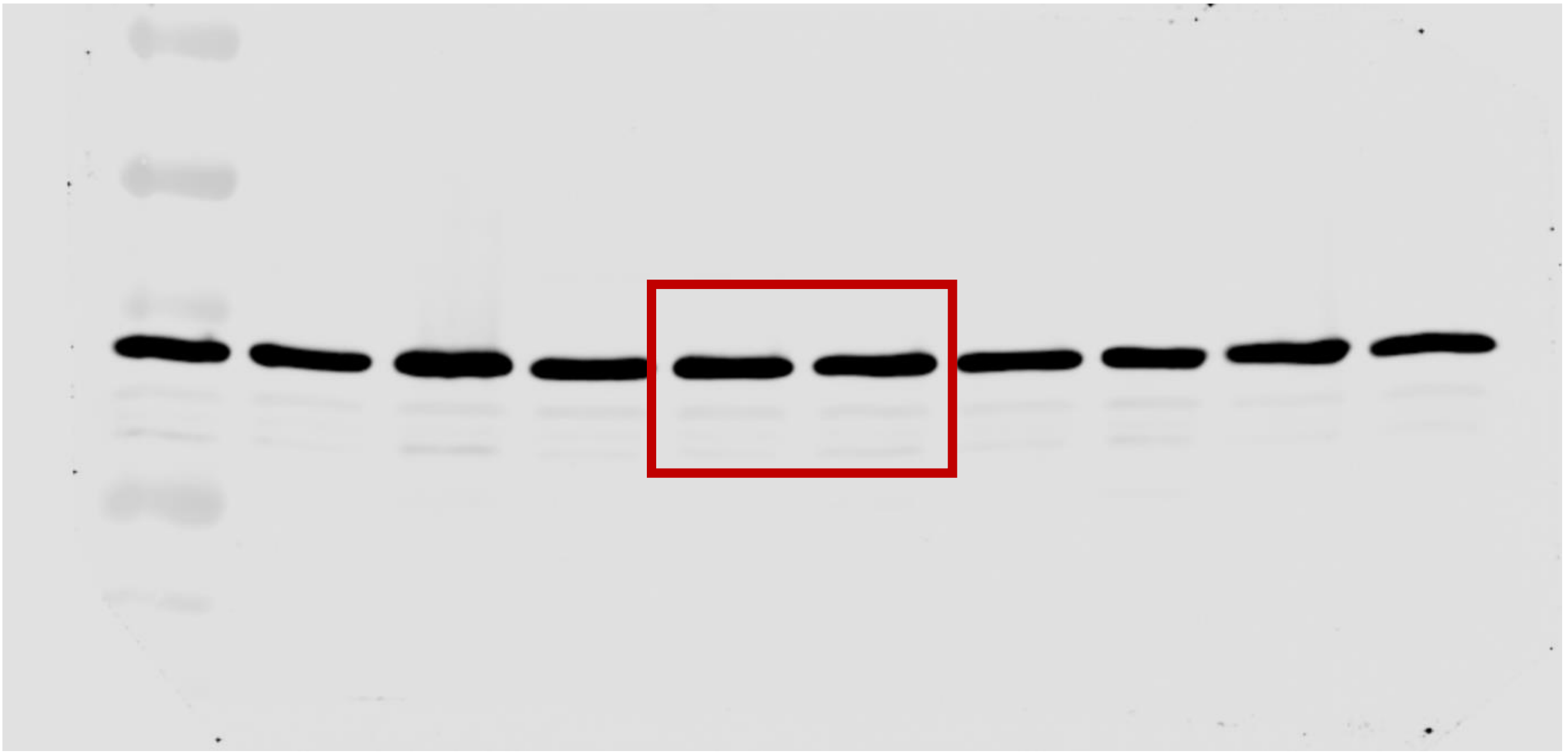

Supplemental Figure 3B. Uncropped/unedited blot images

Left panel: PGE_2 reversal

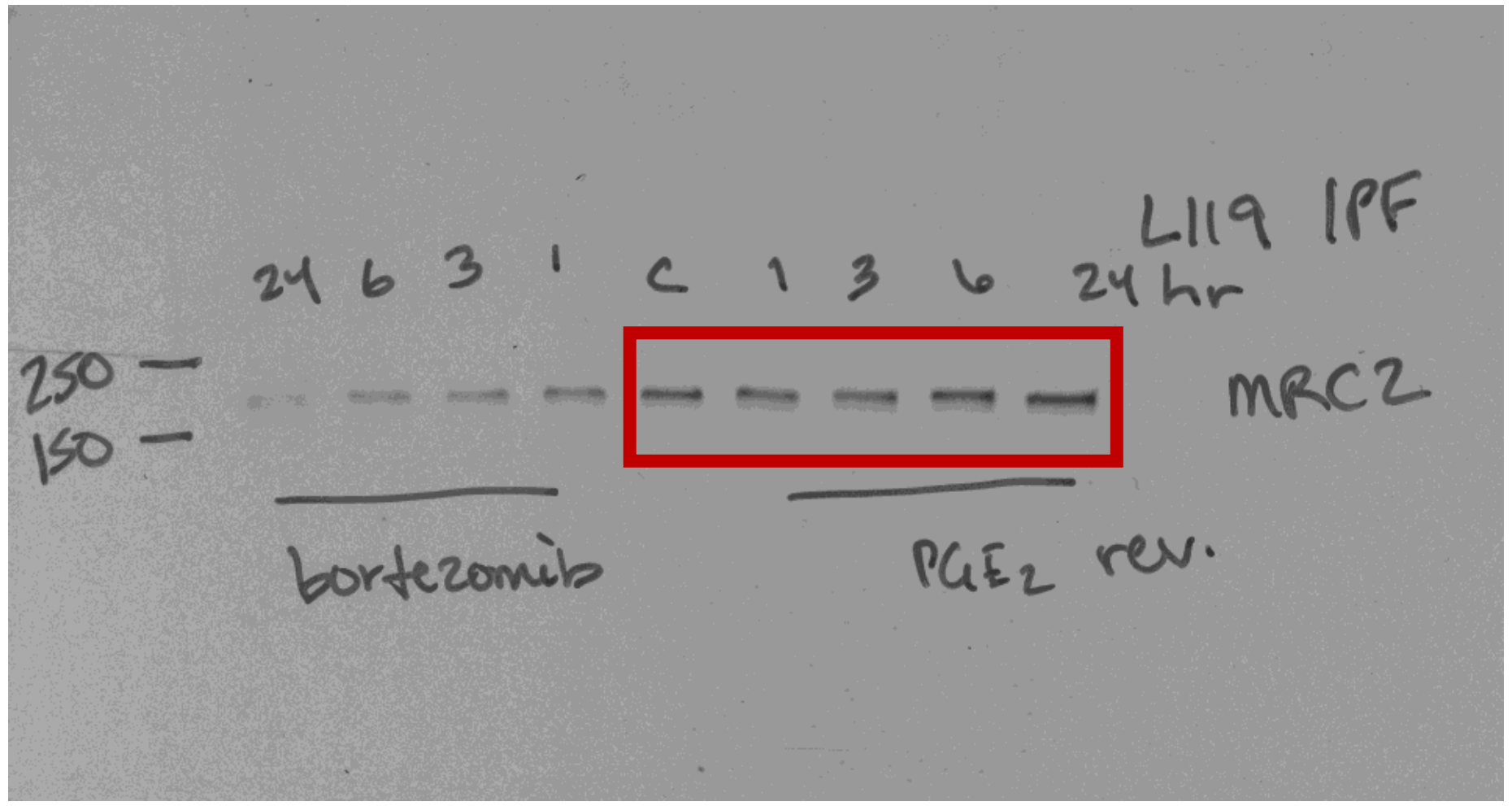

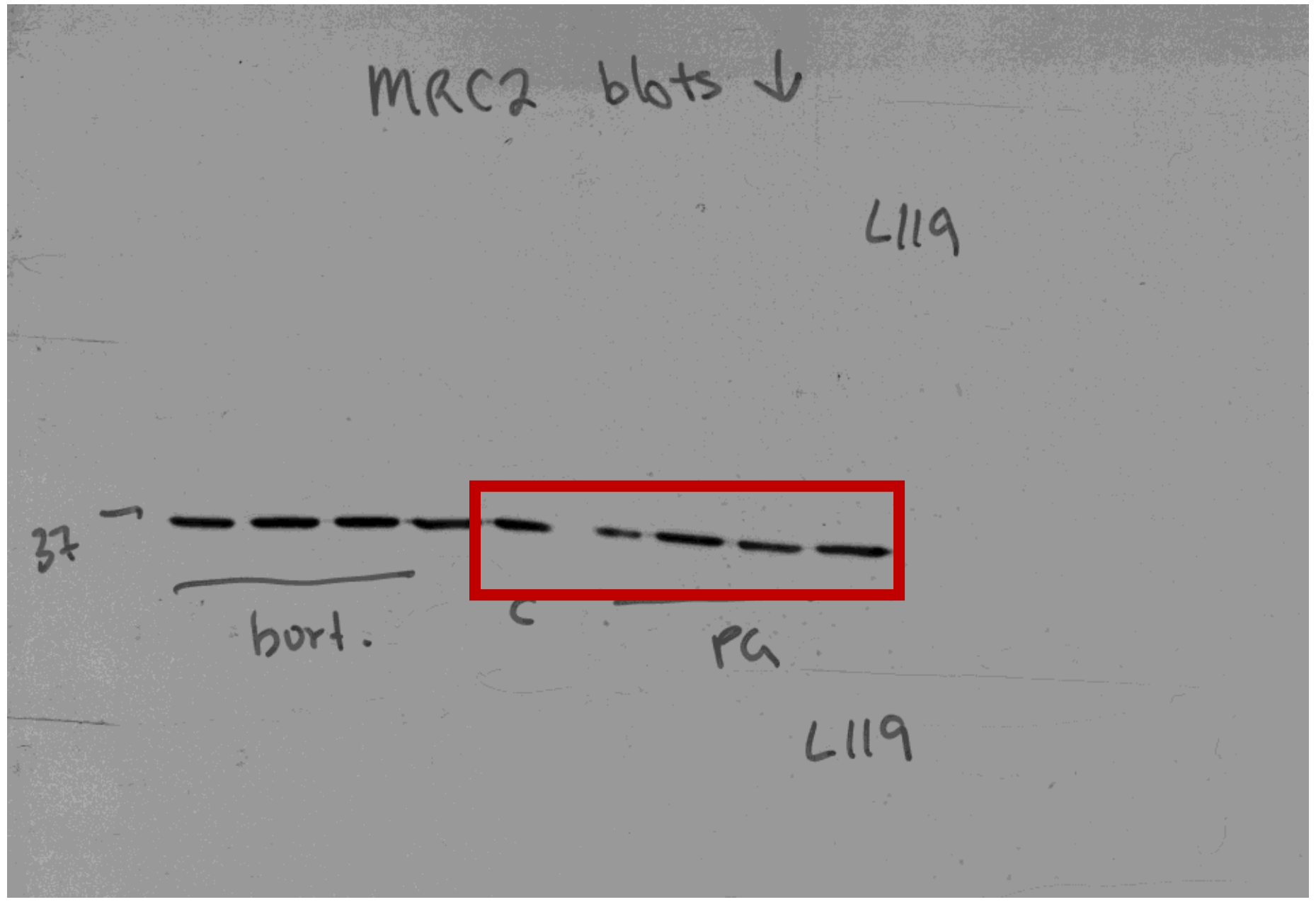

Middle Left panel: Forskolin reversal

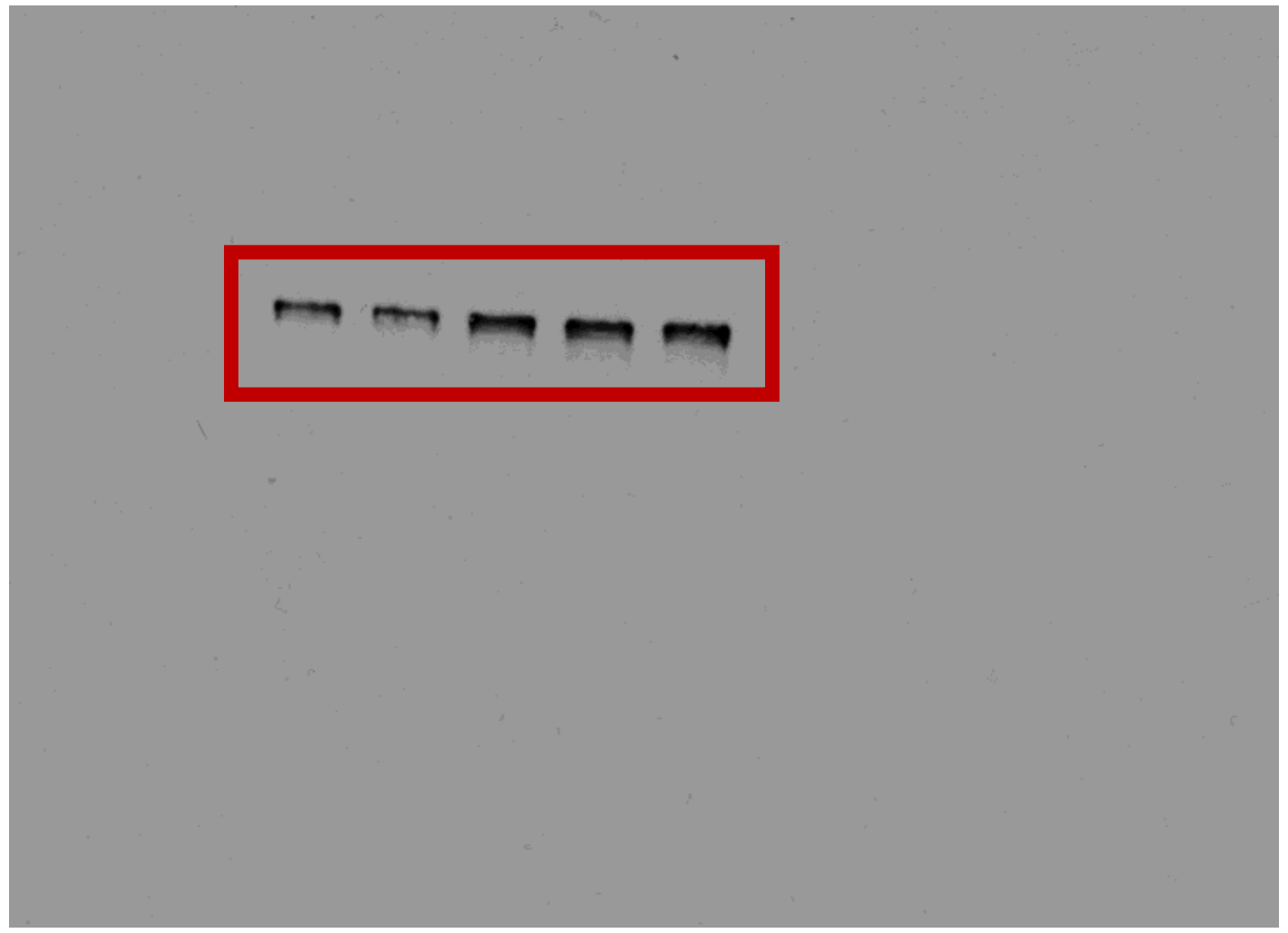

MRC2

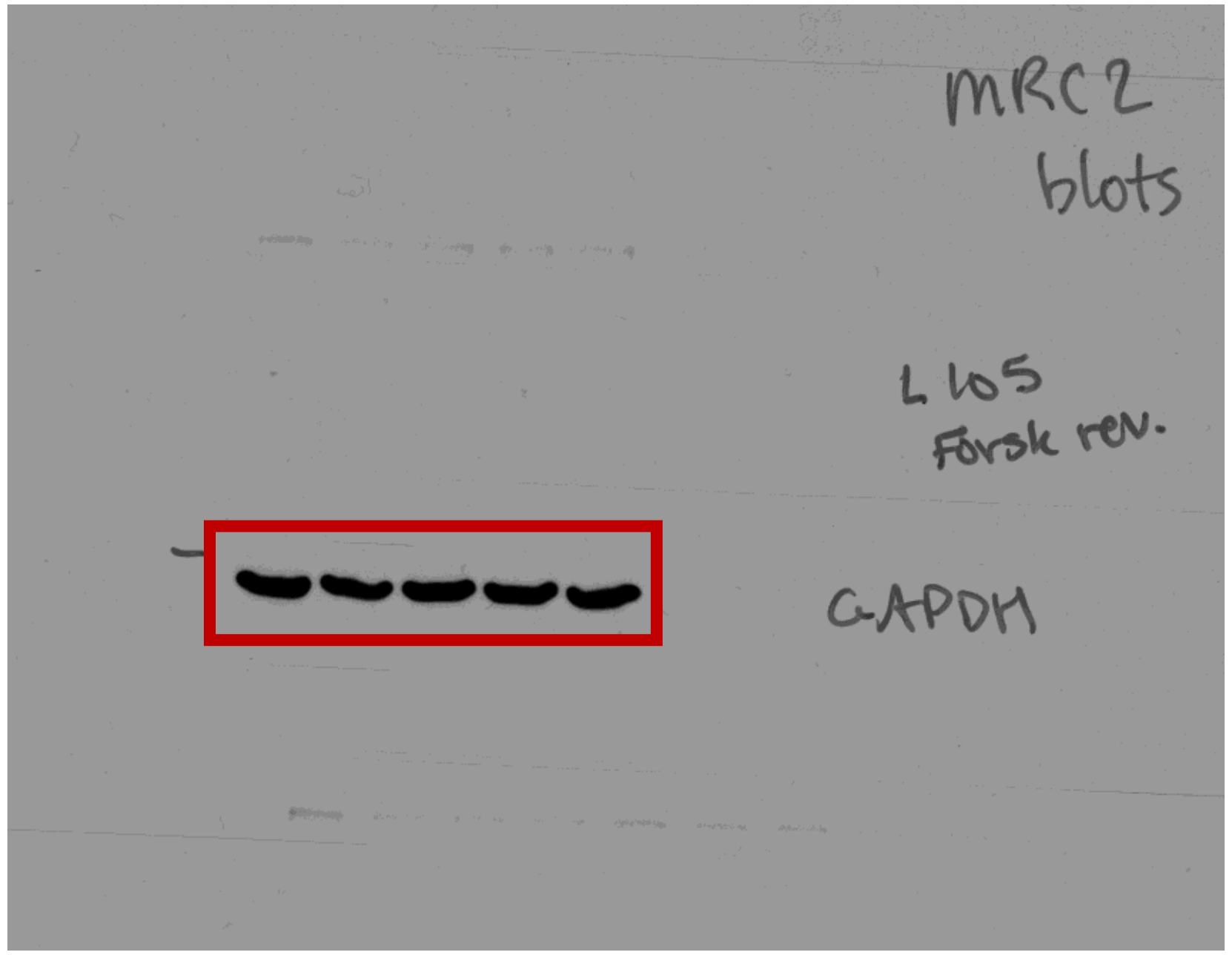

GAPDH

Supplemental Figure 3B. Uncropped/unedited blot images

Middle Right panel: FGF2 reversal

MRC2

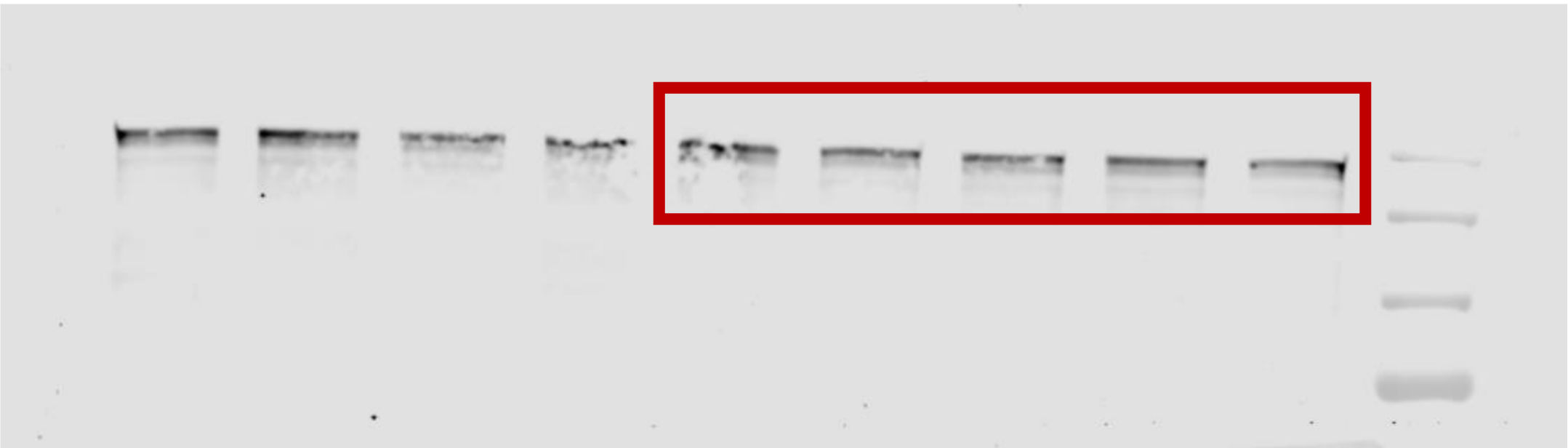

GAPDH

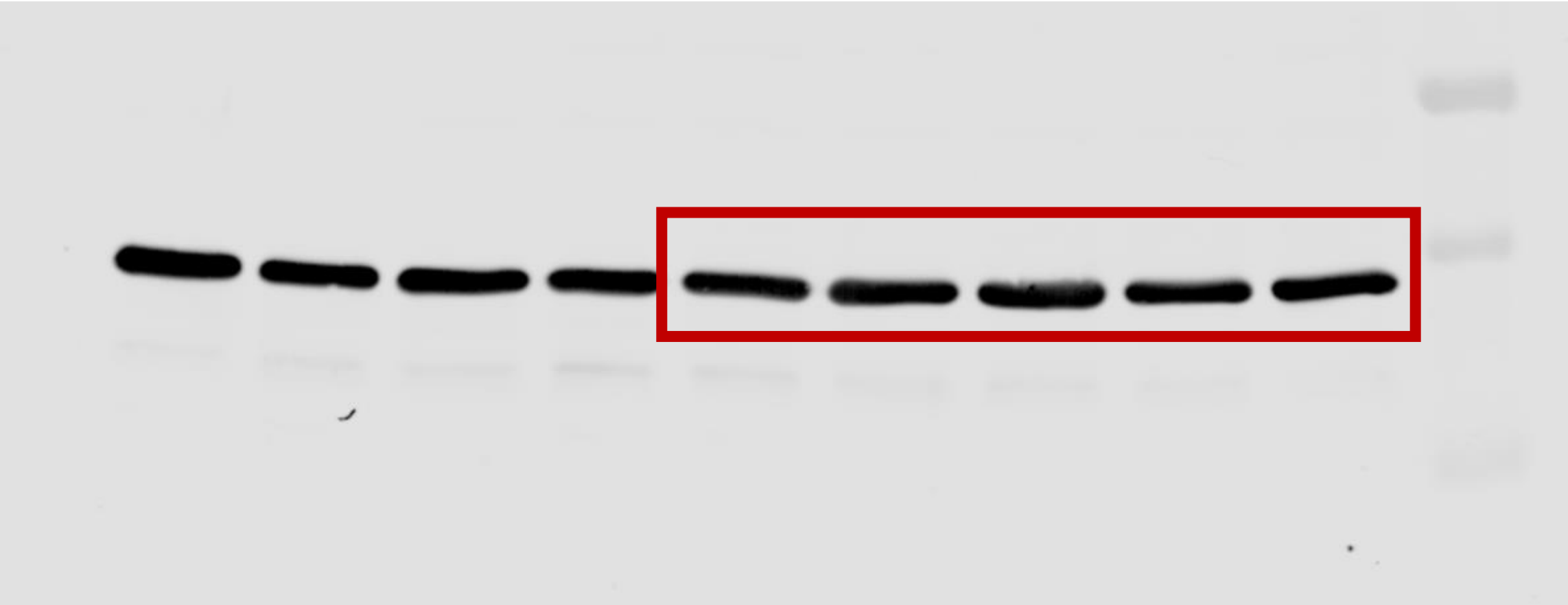

Right panel: Bortezomib reversal

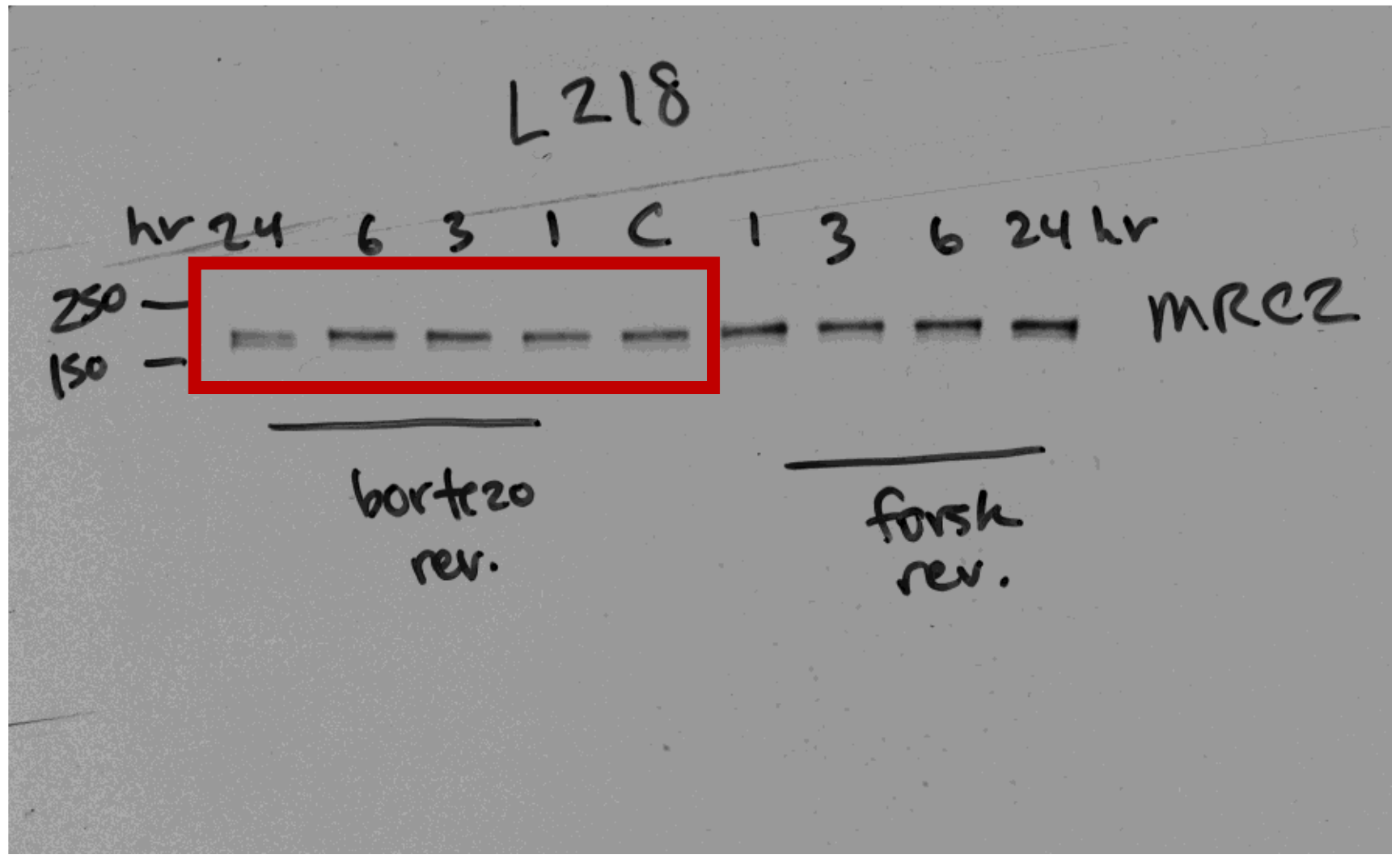

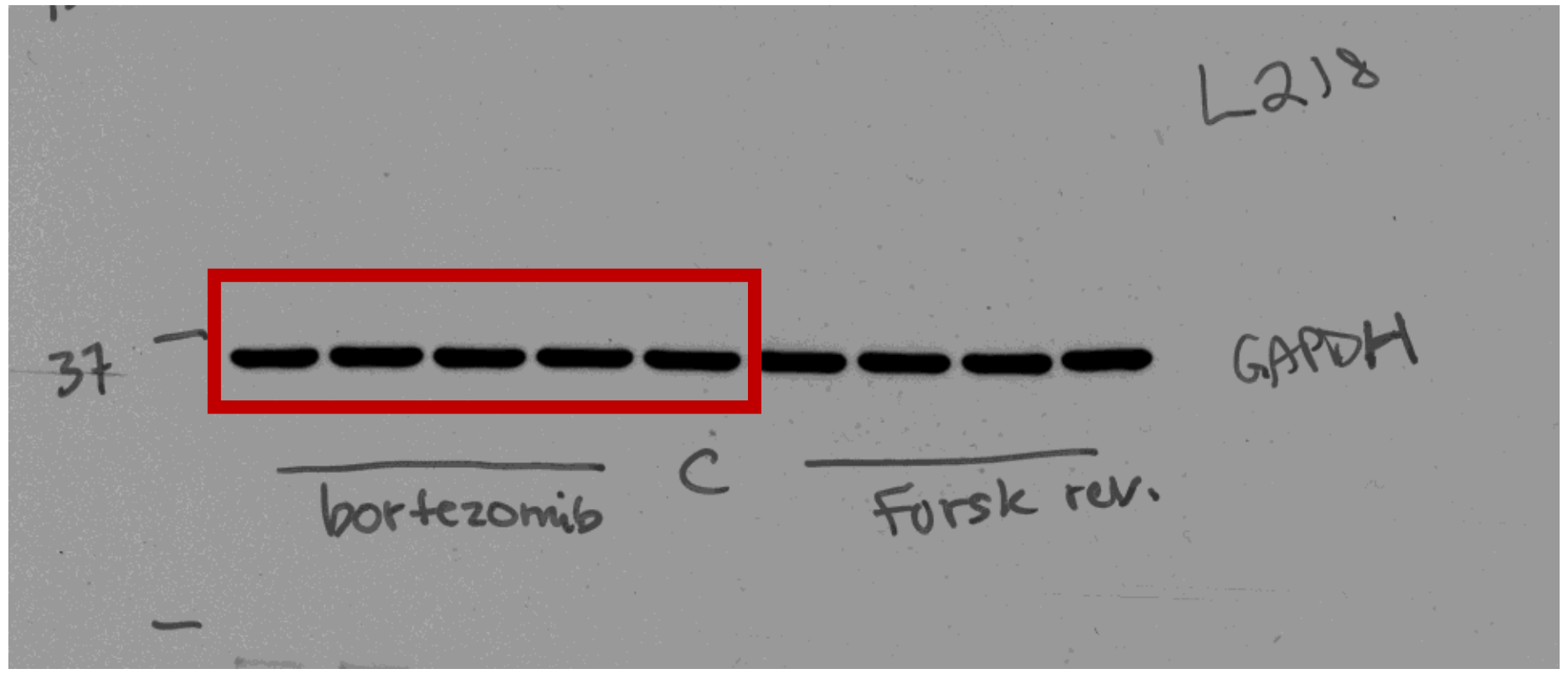

Supplemental Figure 3C. Uncropped/unedited blot images

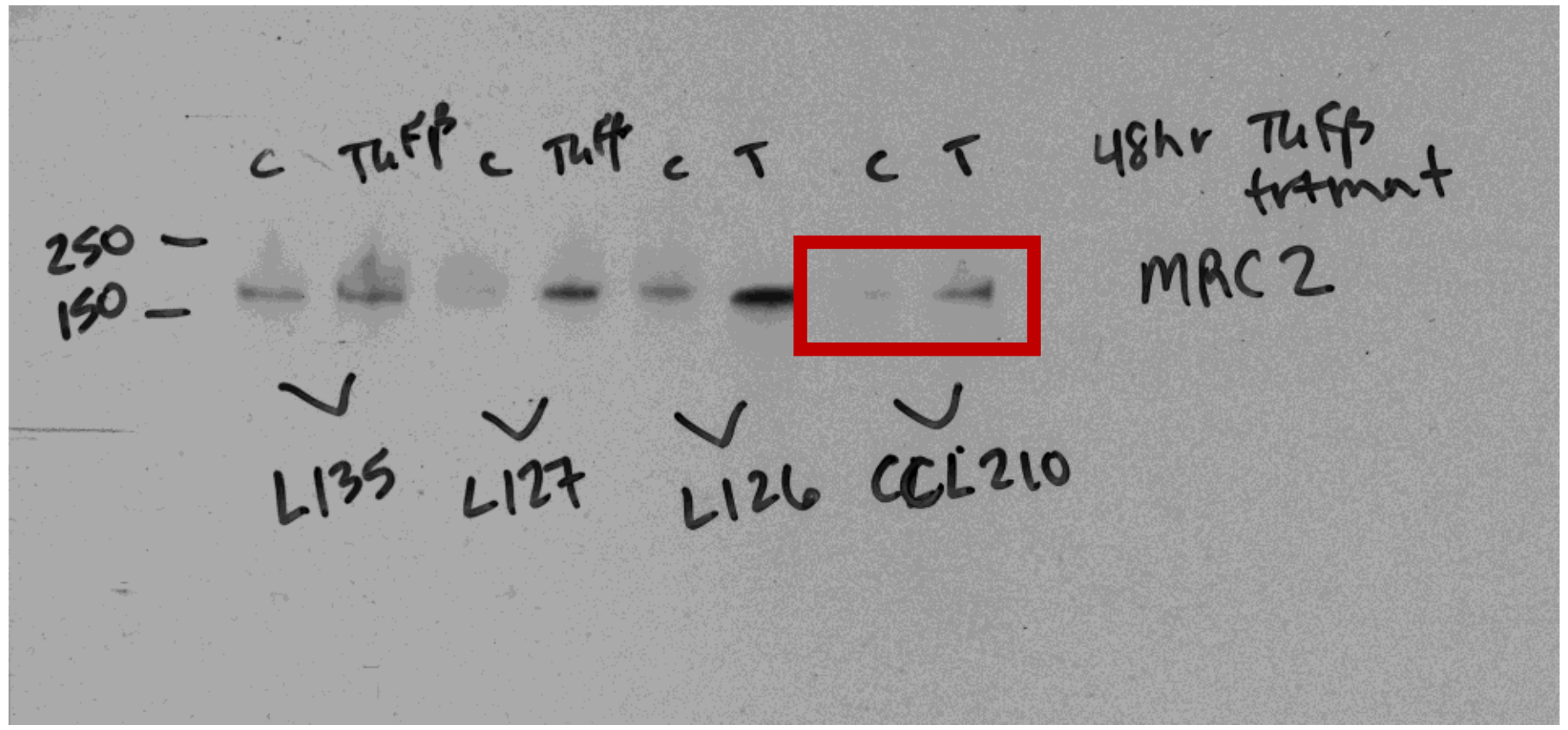

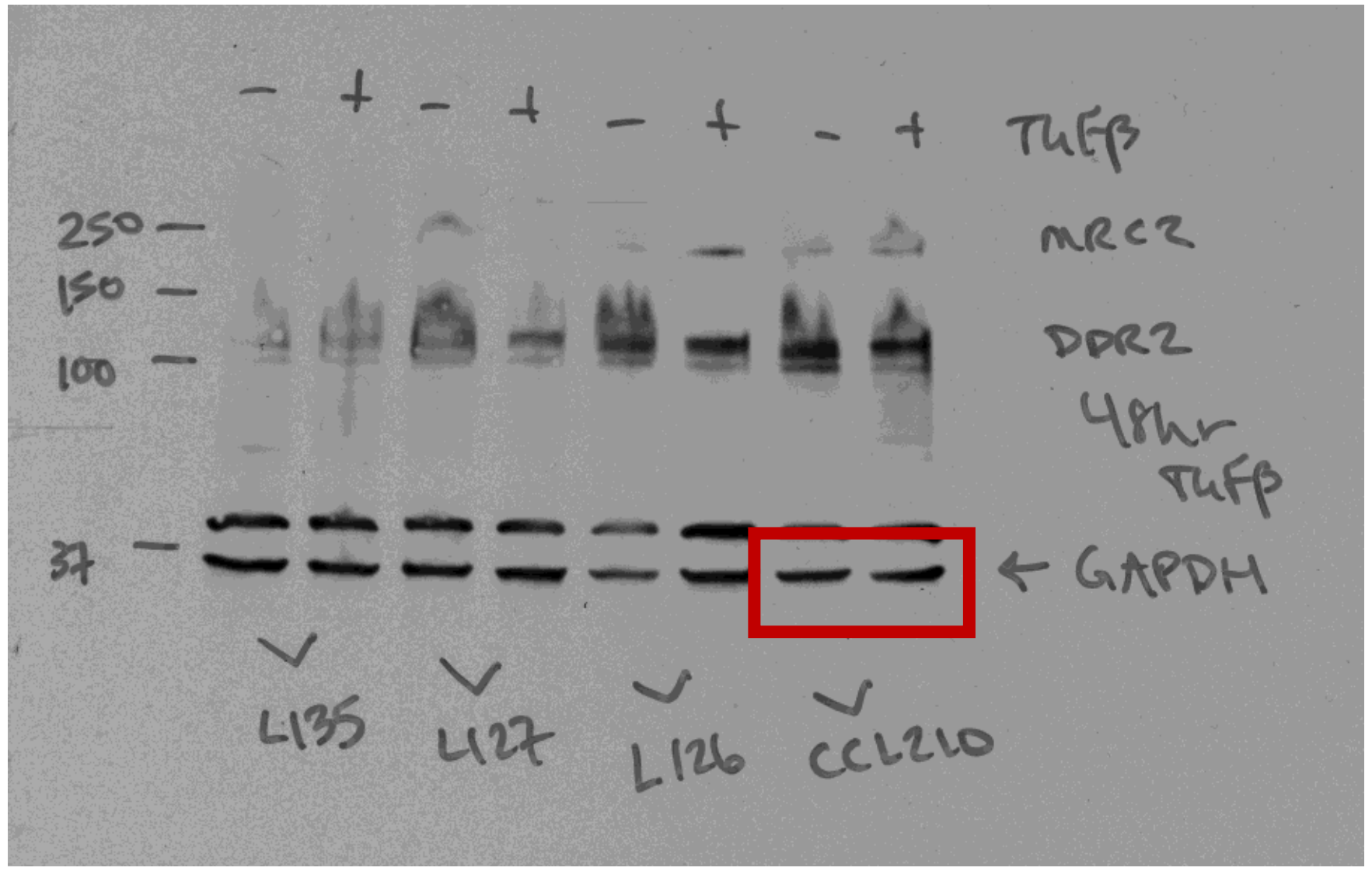

Supplemental Figure 3D. Uncropped/unedited blot images

Left panel: PGE_2 reversal

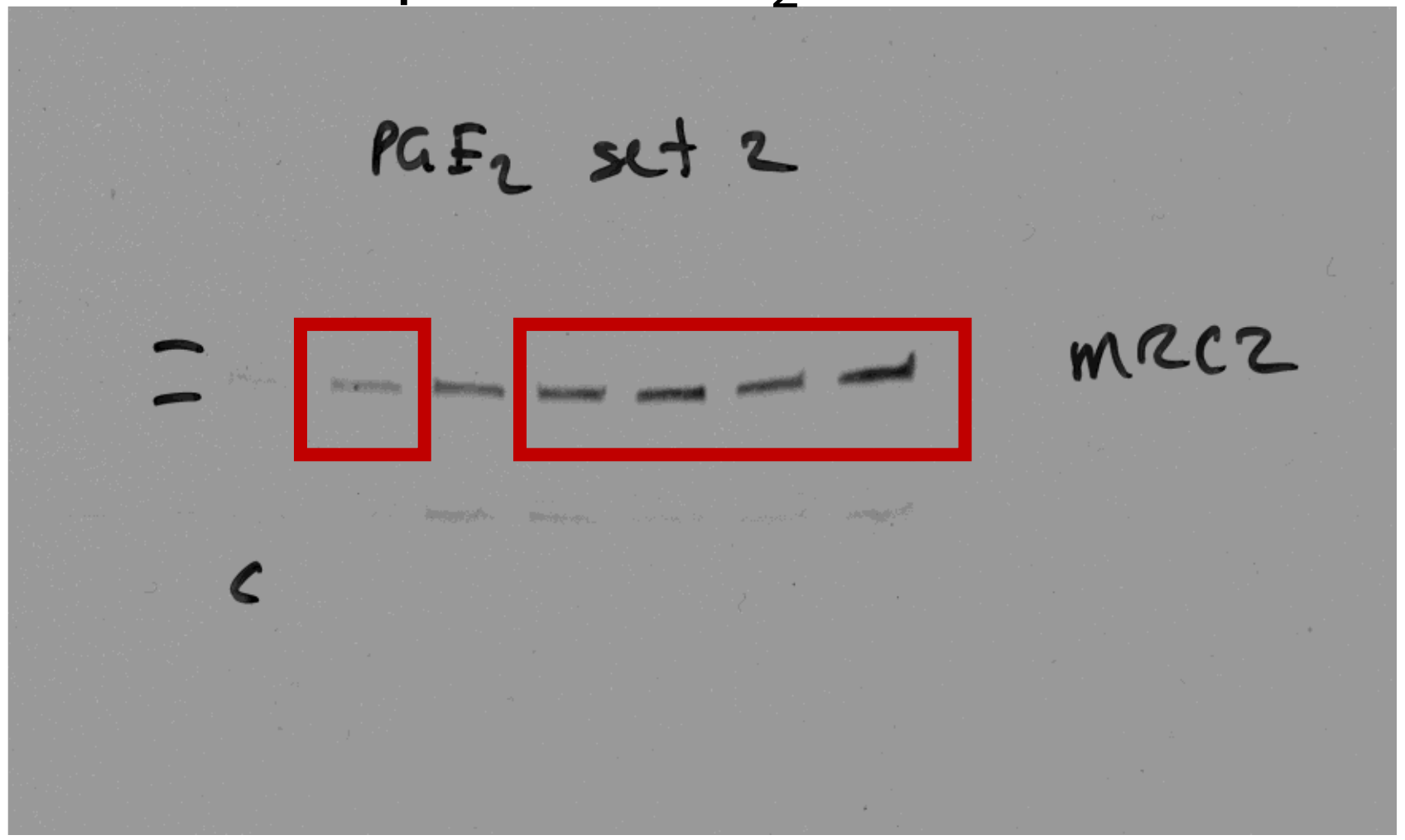

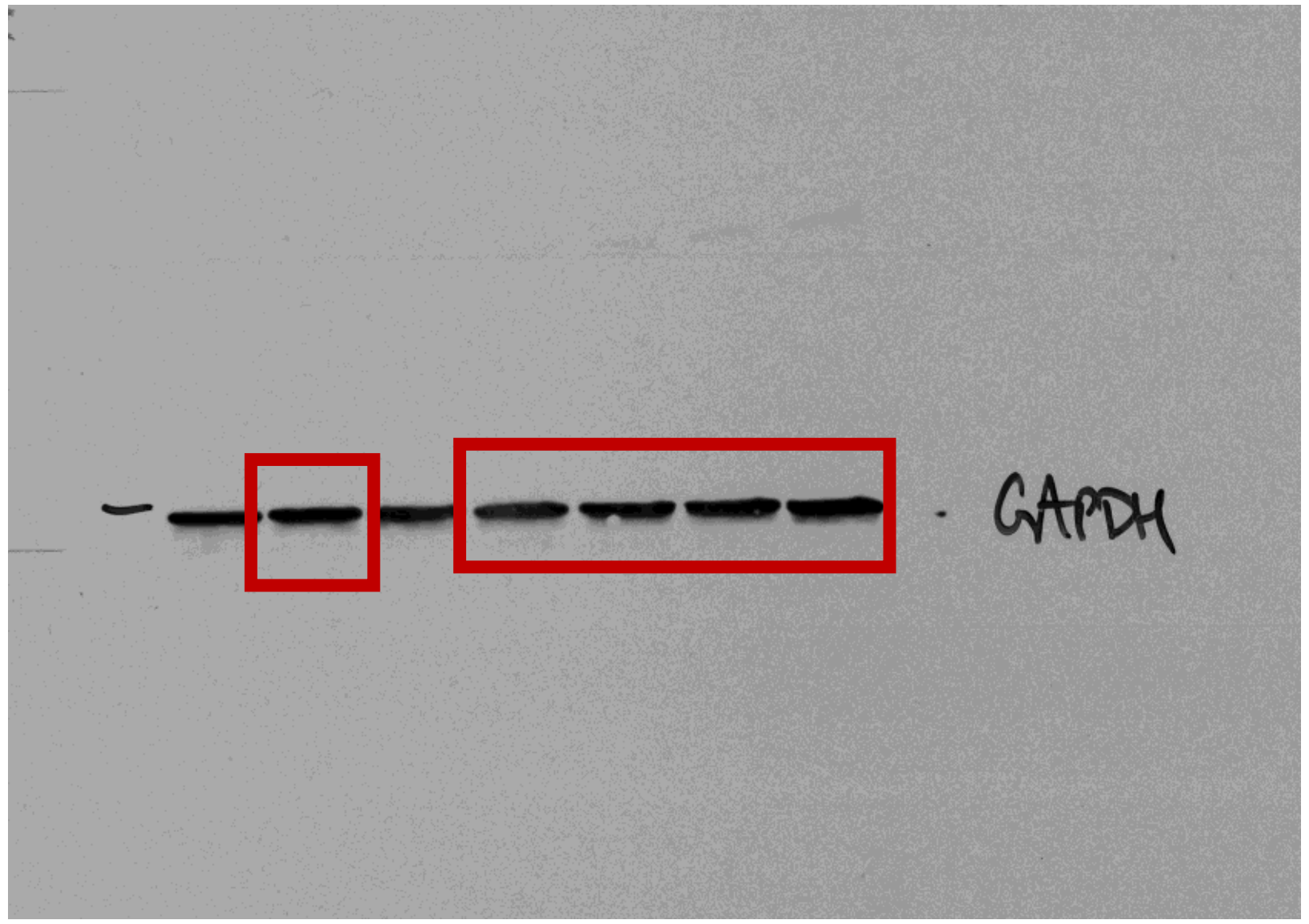

right panel: FGF2 reversal

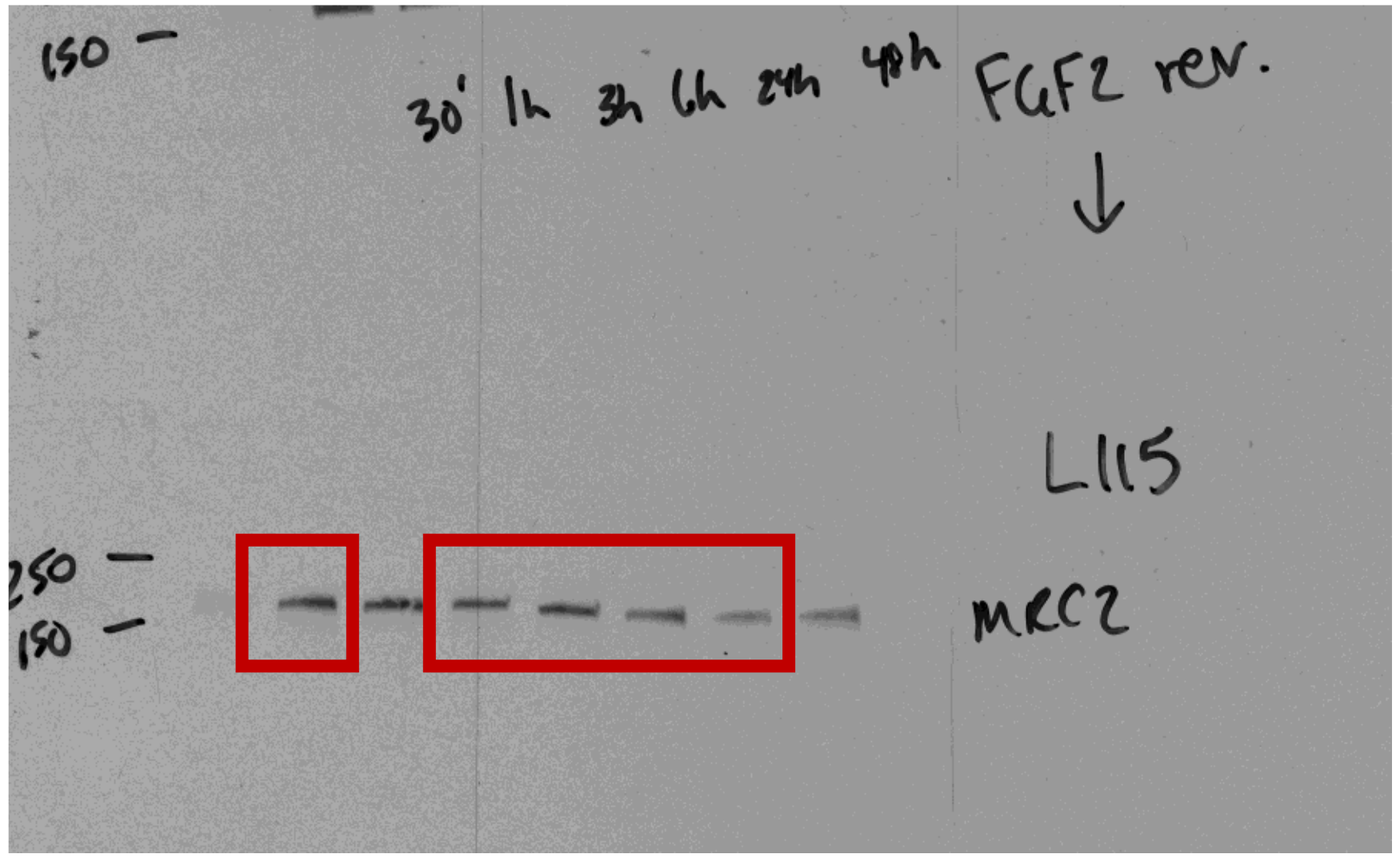

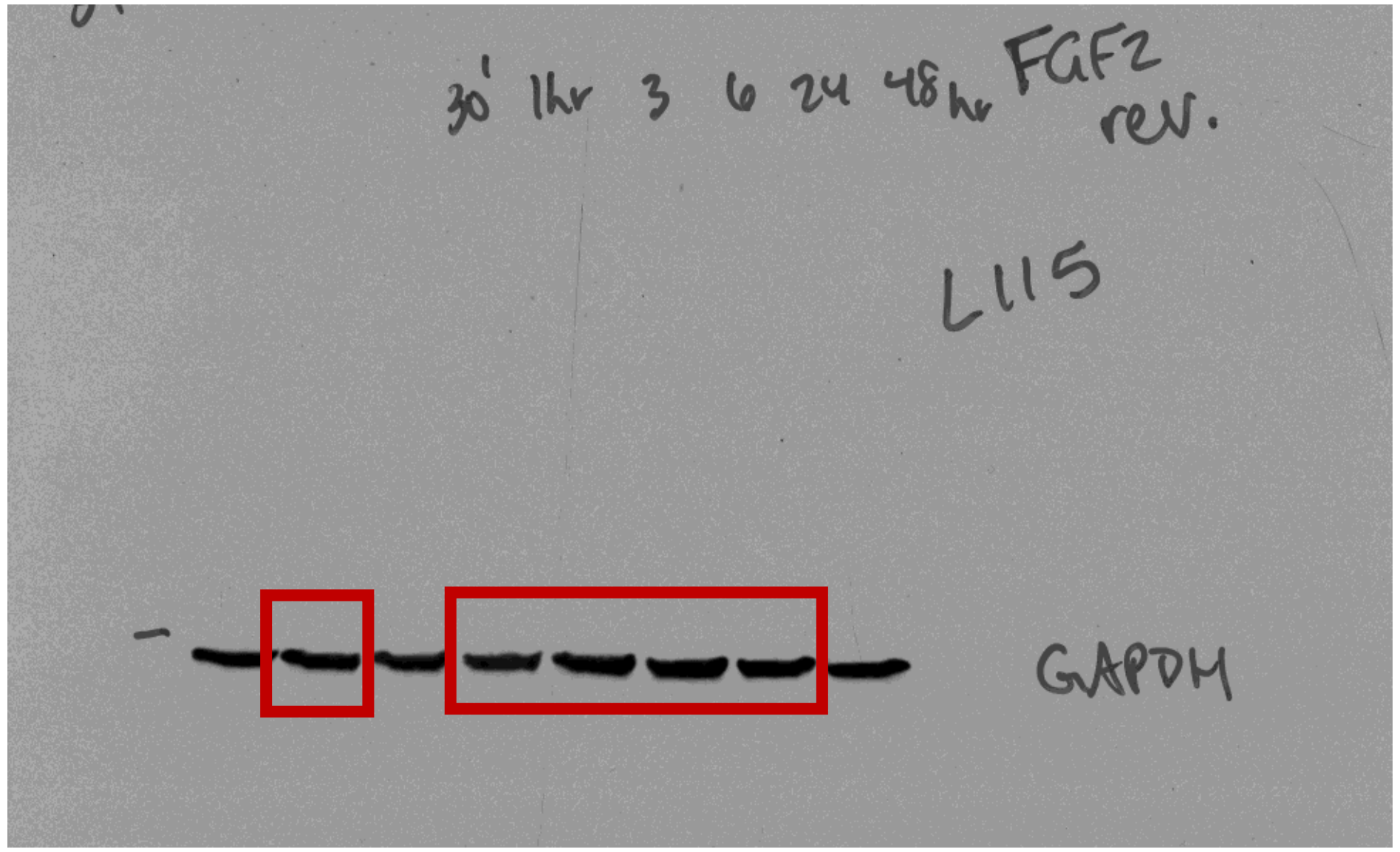

MRC2

GAPDH
